# Percutaneous Left Atrial Appendage Closure: Current Evidence and Procedural Insights

**DOI:** 10.31083/RCM46003

**Published:** 2025-12-22

**Authors:** Mohsen Mohandes, Leydimar Anmad Shihadeh, Alberto Pernigotti, Mauricio Torres, Cristina Moreno, Roberto Bejarano, Francisco Fernández, Jordi Guarinos, Jose Luis Ferreiro

**Affiliations:** ^1^Interventional Cardiology Unit, Cardiology Division, Joan XXIII University Hospital, Pere Virgili Health Research Institute (IISPV), 43005 Tarragona, Spain; ^2^Cardiology Division, Joan XXIII University Hospital, Pere Virgili Health Research Institute (IISPV), 43005 Tarragona, Spain

**Keywords:** atrial fibrillation (AF), left atrial appendage closure (LAAC), oral anticoagulation (OAC), transesophageal echocardiography (TEE), cardiac computed tomography (CCT), transseptal puncture (TSP), direct oral anticoagulant (DOAC), vitamin K antagonist (VKA)

## Abstract

Atrial fibrillation (AF) is the most common sustained arrhythmia and a major cause of cardioembolic stroke, with the left atrial appendage representing the predominant site of thrombus formation. Oral anticoagulation (OAC)—particularly with direct oral anticoagulants—remains the cornerstone of stroke prevention; however, contraindications and bleeding risks limit the use of OAC in selected patients. Percutaneous left atrial appendage closure (LAAC) has emerged as a device-based alternative to oral anticoagulation. Moreover, the indications of LAAC are expanding to include recurrent ischemic stroke despite adequate anticoagulation and patients with advanced chronic kidney disease. Thus, this review synthesizes the current evidence on LAAC and provides a practical, step-by-step procedural roadmap, from preprocedural imaging with transesophageal echocardiography or cardiac computed tomography and anatomical sizing, to transseptal puncture, device selection, deployment, and release criteria, as well as intraprocedural imaging and hemodynamic assessment. Advances in imaging modalities, procedural planning, and device technology have improved both efficacy and safety. However, postprocedural antithrombotic strategies remain heterogeneous and the subject of ongoing clinical trials. Future research is expected to refine patient selection, optimize pharmacotherapy after LAAC, and further define the role of LAAC in the contemporary management of AF.

## 1. Introduction

Atrial fibrillation (AF) is the most common sustained arrhythmia and imposes a substantial burden on healthcare systems. Its prevalence in the general population is estimated at 0.4%–1%, increasing to over 8% among individuals older than 80 years. Moreover, as the population ages, the prevalence of AF is projected to double in the coming decades [1, 2]. In 1909, Welch [3] identified the left atrial appendage (LAA) as the principal site of intracardiac thrombus formation leading to cardioembolic stroke. Since then, the LAA has been recognized as the most frequent site of thrombus development, accounting for approximately 90% of thrombi in patients with non-valvular atrial fibrillation (NVAF), a term that is no longer used in contemporary AF guidelines [4]. Oral anticoagulation (OAC) is recommended for patients with AF and a CHA_2_DS_2_-VA (Congestive heart failure, Hypertension, Age ≥75 years, Diabetes mellitus, Stroke, Vascular disease, Age 65–74 years) score ≥2 (Class I, Level of Evidence C) and should be considered for those with a score of 1 (Class IIa, Level of Evidence C) [1]. Direct oral anticoagulants (DOACs) have demonstrated at least noninferior efficacy compared with warfarin to prevent thromboembolism, with the added advantage of reducing intracranial hemorrhage [5, 6, 7, 8]. However, at standard doses, DOACs have not consistently shown significant reductions in other major bleeding events and, in some studies, have been associated with an increased risk of major gastrointestinal bleeding [9]. This risk appears to be more pronounced with dabigatran and rivaroxaban compared with conventional anticoagulation [10]. Before initiating OAC, an individualized assessment of thromboembolic and bleeding risk is essential. Modifiable bleeding risk factors—such as uncontrolled hypertension or concomitant antiplatelet therapy—should be addressed to enhance treatment safety. While many bleeding risk factors are manageable and do not constitute absolute contraindications to OAC in AF, there are a few absolute contraindications, including primary intracranial tumors, intracerebral hemorrhage related to cerebral amyloid angiopathy [11, 12], and certain hereditary hematological disorders such as severe hemophilia (factor VIII/IX <20%) [13].

Left atrial appendage closure (LAAC) can be performed either surgically or percutaneously. Surgical LAAC is a well-established procedure for which several techniques have been described in the literature [14]. It carries a Class I recommendation (Level of Evidence C) for concomitant use during open-heart surgery [1]. However, the focus of this review is LAAC performed via a percutaneous approach. Percutaneous LAAC is a device-based intervention designed to occlude the LAA and represents an alternative for patients with AF who are not suitable for long-term OAC. The utilization of LAAC has grown substantially in recent years, driven by technological innovation and expanding procedural expertise. In this review, we summarize the current evidence supporting LAAC, discuss clinical scenarios in which it may be particularly beneficial, and highlight key technical and procedural considerations.

## 2. Left Atrial Appendage Anatomy

**Fig. 1.**
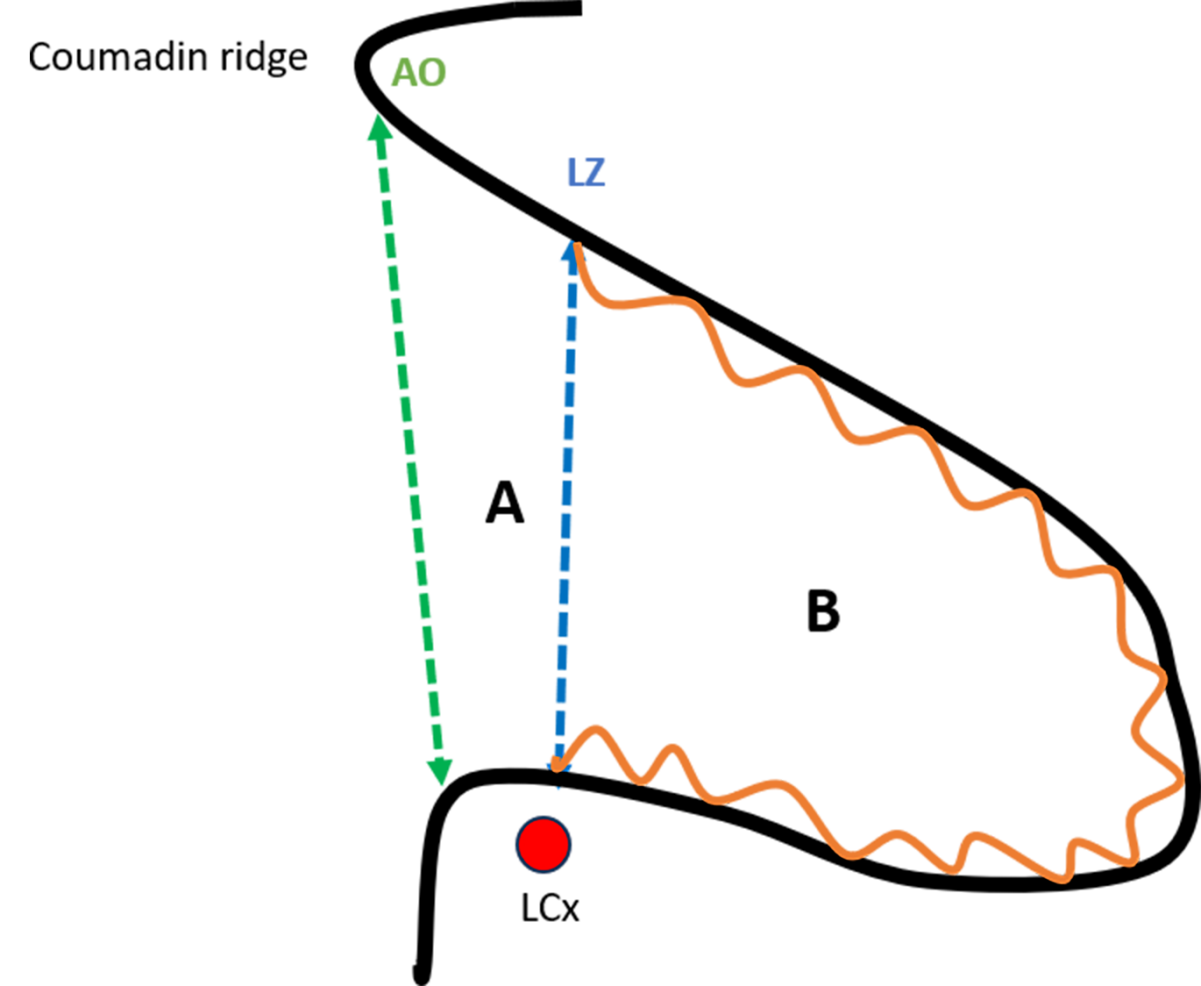
**Anatomy of the left atrial appendage (LAA)**. (A) Proximal neck. (B) Functional (trabeculated) portion. AO, anatomic ostium; LZ, landing zone; LCx, left circumflex artery.

The LAA is a complex, highly variable structure located on the anterolateral portion of the left atrium (LA). Anatomically, it can be divided into two main regions: a proximal neck portion, which includes the anatomical ostium—typically ovoid in shape—and a distal trabeculated portion, which constitutes the functional body of the LAA (Fig. 1). The smooth-walled neck portion is embryologically derived from the pulmonary veins, whereas the trabeculated functional portion originates from the primordial LA. This latter region is the principal site of thrombus formation. The ostium of the functional LAA is typically located at the level of the left circumflex artery (LCx). During LAAC procedures, this region is commonly referred to as the landing zone [15, 16].

**Fig. 2.**
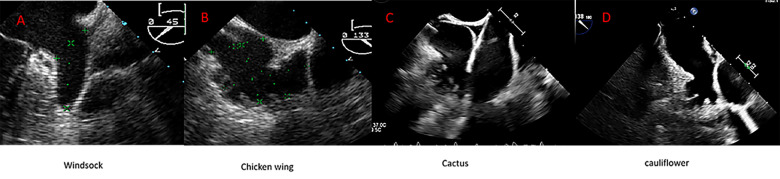
**Anatomic variant of the left atrial appendage (LAA) depicted by transesophageal echocardiography (TEE)**. (A) Windsock; (B) Chicken wing; (C) Cactus; (D) Cauliflower.

The LAA is separated from the left superior pulmonary vein (LSPV) by the Coumadin ridge, an endocardial fold that externally corresponds to the ligament of Marshall. The LAA may have one or more lobes or protrusions, which are extensions of its functional portion [17]. Based on cardiac computed tomography (CCT) findings, Wang *et al*. [18] classified the LAA into four main morphologies: windsock, cauliflower, cactus, and chicken wing (Fig. 2). The authors also described the spatial relationship between the LAA and the LSPV, categorizing it as high type (superior to the LSPV), a mid-type (parallel to the LSPV), or a low type (inferior to the LSPV). Among these, the chicken-wing morphology is the most common variant and is generally considered the most challenging for percutaneous closure due to its broad ostium and shallow depth. Conversely, the cauliflower morphology has been most frequently associated with embolic events [16, 19].

Of note, the current morphological classifications have demonstrated limited predictive value for procedural complexity and clinical outcomes in LAAC [18]. Therefore, additional anatomical and functional parameters should be considered during preprocedural planning, as discussed later in this review.

## 3. Percutaneous Left Atrial Appendage Closure: A Review of the Published Clinical Studies

During the era of vitamin K antagonists (VKAs), two randomized controlled trials—PROTECT AF and PREVAIL [20, 21]—compared percutaneous LAAC using the Watchman device with warfarin in patients with NVAF. The primary objective of both studies was to demonstrate the noninferiority of LAAC with respect to a composite endpoint comprising all-cause stroke, cardiovascular (CV) or unexplained death, and systemic embolism (SE). In both trials, an absolute contraindication to warfarin was an exclusion criterion. Although the PROTECT AF trial met the noninferiority criterion for the primary composite endpoint, procedure-related ischemic strokes occurred more frequently in the device group than in the VKA group—a finding that was also observed in the PREVAIL trial. However, in PREVAIL, the rate of the second co-primary endpoint—stroke or SE occurring more than 7 days after randomization—was similar between the groups, thereby meeting the noninferiority criterion (Table 1). The first co-primary endpoint in PREVAIL did not meet the criteria for noninferiority; however, the event rate in the warfarin group (0.7% per year for ischemic stroke) was substantially lower than anticipated, making it difficult to definitively establish noninferiority. In fact, the rate of stroke or SE in the warfarin group was substantially lower than in other trials comparing warfarin with DOACs for stroke prevention in patients with AF, such as RE-LY, ARISTOTLE, and ROCKET AF, which reported an ischemic stroke rate in the warfarin arm of 1.7%, 1.6%, and 2.2% per year, respectively [5, 7, 8]. Additionally, the rate of serious pericardial effusion in the device group was 4.8%, compared with 0% in the warfarin group. These complications were likely related to limited operator experience at the time and subsequently decreased in later studies, as discussed below.

**Table 1.** **Studies on percutaneous left atrial appendage closure**.

Study	Device/comparator	Endpoints	Outcomes/results
Protect AF (RT)	Watchman/Warfarin	Stroke, SE or CV/unexplained death	NI met
Prevail (RT)	Watchman/Warfarin	1°: Stroke, SE or CV/unexplained death	NI not met
		2°: Stroke or SE >7 days	NI met
Holmes DR *et al*. [22] (MA)	Watchman/Warfarin	- Hemorrhagic stroke, CV death, Non-procedure major bleeding	LAAC better
		- Ischemic stroke	Warfarin better
Reddy VY *et al*. [23] (MA)	Watchman/Warfarin	- Stroke/SE/CV death	Similar
		- Hemorrhagic stroke, disabling stroke CV/unexplained death, all cause death and post-procedure bleeding	LAAC better
ASAP (Regitry)	LAAC (watchman) ineligible for warfarin	Efficacy outcome (IS, HS, SE CV death)	IS: 1.7%/yr
			HS: 0.6%/yr
EWOLUTION (Regitry)	LAAC (Watchman); 73% unsuitable for OAC	Data in routine practice from a prospective multicenter registry	IS: 1.1%
PRAGUE 17 (RT)	DOAC vs. LAAC in high-risk patients	Stroke, TIA; SE CV death, clinically significant bleeding, procedure/device related complications	NI
Amulet IDE (RT)	Amulet/Watchman	Safety: procedure-related complications, all-cause death or major bleeding at 12 months	Similar
		Effectiveness: IS or SE at 18 months, and rate of LAA occlusion at 45 days	Similar
The SWISS-APERO (RT)	Amulet/Watchman	1°: endpoint: justified crossover to a nonrandomized device or residual LAA patency at 45 days	Amulet: no superior
		2°: endpoint: procedural complications, device related thrombus and peridevice leak and clinical outcomes at 45 days	Major complications: higher with Amulet
			Clinical outcomes: similar
Amplatzer Amulet (Registry)	Amulet	The periprocedural and early clinical/TEE results up 3 months	Successful implantation: 99%
			MAEs: 3.2%
			Adequate occlusion: 98.2%
Pinnacle FLX (prospective single arm study)	Watchman FLX	- Safety: death, IS, SE or device/procedure related events requiring cardiac surgery	Performance goal met
		- Effectiveness: effective closure at one year follow-up	Performance goal met
SURPASS (observational registry)	Watchman FLX	Safety: death, IS, SE or device/procedure related events requiring cardiac surgery or major endovascular intervention	Outcomes similar to Pinnacle FLX

CV, cardiovascular; DOAC, direct oral anticoagulant; HS, hemorrhagic stroke; IS, ischemic stroke; LAAC, left atrial appendage closure; MA, metanalysis; NI, non-inferiority; OAC, oral anticoagulation; RT, randomized trial; SE, systemic embolism; TIA, transient ischemic attack; Yr, year; LAA, left atrial appendage; TEE, transesophageal echocardiography; MAEs, Major Adverse Events; AF, atrial fibrillation.

Holmes DR *et al*. [22] conducted a meta-analysis including patients from the PROTECT AF and PREVAIL trials, as well as their respective registries (the Continued Access to PROTECT AF [CAP] registry and the Continued Access to PREVAIL [CAP2] registry), with a mean follow-up of 2.69 years. The authors reported significantly fewer hemorrhagic strokes, CV or unexplained deaths, and non-procedure-related major bleeding events in the device group compared with the warfarin group. In contrast, ischemic strokes were more frequent in the device group; however, when procedure-related strokes—those occurring within the first 7 days post-randomization—were excluded, this difference was no longer statistically significant.

Reddy VY *et al*. [23] conducted a meta-analysis of the PROTECT AF and PREVAIL trials with a 5-year follow-up. Their study revealed that the composite primary endpoint—including stroke, SE, or CV death—was similar between the LAAC and warfarin groups. Although the incidence of ischemic stroke or SE was numerically higher in the device group, the difference was not statistically significant. Moreover, the rates of hemorrhagic stroke, disabling or fatal stroke, CV or unexplained death, all-cause mortality, and postprocedural bleeding favored the LAAC group.

Several methodological concerns have been raised regarding the PROTECT AF and PREVAIL trials. Unlike randomized trials comparing non-VKAs (i.e., DOACs) with warfarin—which enrolled tens of thousands of patients—PROTECT AF and PREVAIL included only 730 and 382 participants, respectively. These small sample sizes reduce the statistical power to detect differences in infrequent outcomes, such as embolic events or major bleeding in patients with AF. Moreover, the inclusion of CV or unexplained death as part of the composite primary endpoint is debatable, given that these outcomes are less likely to be directly influenced by either treatment strategy [24, 25]. Finally, it is worth noting that these initial randomized trials were conducted in patients without contraindications to OAC, which contrasts with the main indication for LAAC in current practice—namely, patients who are unsuitable for long-term anticoagulation. In fact, in both studies, warfarin was administered for 45 days post-LAAC in the device group.

ASAP study [26] was a multicenter, prospective, non-randomized registry involving patients with AF who were ineligible for warfarin therapy and underwent LAAC with the Watchman device. The most common reason for warfarin ineligibility was a history of hemorrhagic events or a bleeding tendency, accounting for 93% of cases. Following LAAC, patients received dual antiplatelet therapy (DAPT)—aspirin plus a thienopyridine—for 6 months, followed by lifelong aspirin monotherapy. The mean follow-up duration was 14.4 ± 8.6 months, during which the annual rate of ischemic stroke was 1.7%, substantially lower than the expected rate of 7.3% based on the patients’ CHADS_2_ scores. Of note, serious procedure- or device-related adverse events occurred in 8.7% of patients, including pericardial effusion in 3.3% of patients (with 1.3% requiring pericardiocentesis).

The EWOLUTION registry [27] is a prospective, multicenter study designed to provide real-world data on LAAC. A total of 1025 patients underwent LAAC with the Watchman device, of whom 73% were deemed unsuitable for OAC. Device implantation was successful in 98.5% of cases. At the 1-year follow-up, the observed ischemic stroke rate was 1.1%, notably lower than the expected rate of 7.2% based on the CHA_2_DS_2_-VASc score.

**Fig. 3.**
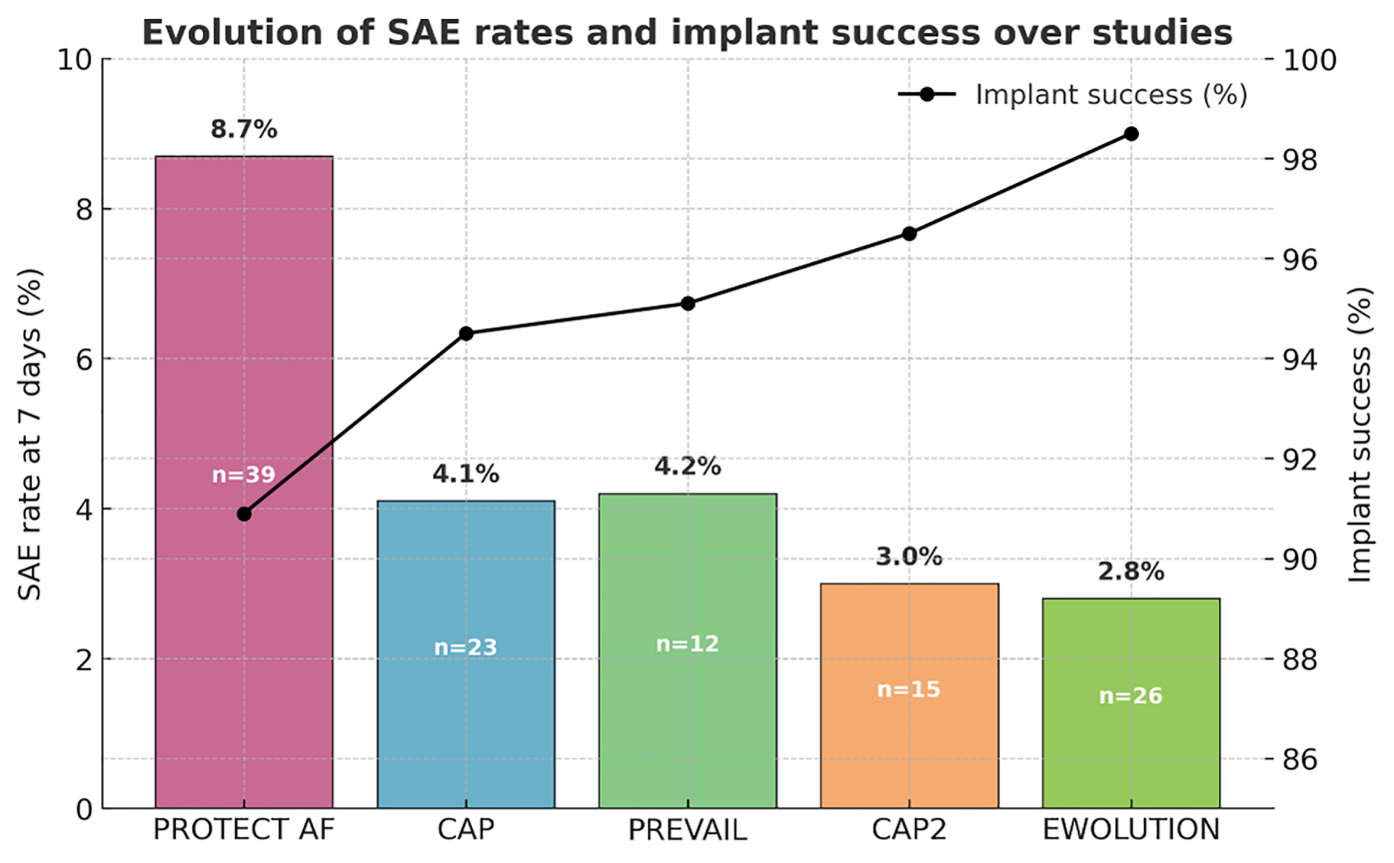
**Evolution of the serious adverse event (SAE) rates within 7 days and implant success across the PROTECT AF, CAP, PREVAIL, CAP2, and EWOLUTION studies**. CAP, the Continued Access to PROTECT AF; CAP2, the Continued Access to PREVAIL.

Within the first 7 days after the procedure, serious procedure- or device-related adverse events occurred in 2.8% of patients, including four deaths—one of which was attributed to cerebral air embolism on the day of the procedure. Fig. 3 illustrates the evolution of serious adverse events within the first 7 days across the PROTECT AF, CAP, PREVAIL, CAP2, and EWOLUTION studies, demonstrating a significant reduction over time. This trend likely reflects growing operator experience and procedural refinement. Notably, device implantation success has increased from 90.9% in the PROTECT AF study to 98.5% in the EWOLUTION registry.

The PRAGUE 17 [28] trial was a multicenter, randomized study designed to compare DOAC therapy with LAAC in patients with AF who had a prior history of bleeding requiring intervention or hospitalization, a history of cardioembolic events despite anticoagulation, and/or a CHA_2_DS_2_-VASc score ≥3 with a HAS-BLED score >2. Apixaban was the most frequently used DOAC, accounting for 95.5% of cases. The devices used in this trial were Amulet (61.3%), Watchman (35.9%), and Watchman FLX™ (2.8%). LAAC was successfully performed in 96.8% of attempted procedures. After a median follow-up of 19.9 months, the primary outcome—comprising stroke or transient ischemic attack (TIA), SE, clinically significant bleeding, CV death, and periprocedural or device-related complications (applicable only to the LAAC group)—was noninferior in the LAAC group compared with the DOAC group, with no significant differences in the individual components of the endpoint. The rate of major LAAC-related complications was 4.5%, including one device-related death, one procedure-related death, two cases of late pericardial effusion, two vascular complications, and one device embolization.

Following LAAC, the recommended antithrombotic regimen consisted of clopidogrel 75 mg/day plus aspirin 100 mg/day for 3 months. If transesophageal echocardiography (TEE) at 3 months ruled out device-related thrombus (DRT) and a peridevice leak ≥5 mm, then clopidogrel was discontinued and aspirin was continued indefinitely. Notably, the 4-year follow-up of the PRAGUE 17 study confirmed the noninferiority of LAAC in terms of CV, neurological, and bleeding events, with the additional benefit of a significant reduction in non-procedural bleeding events as an individual component [29].

The Amulet IDE trial [30] was a randomized controlled study designed to compare the effectiveness and safety of LAAC using the Amulet device versus the Watchman device. A total of 1878 patients with AF and an increased risk of stroke were randomly assigned (1:1) to undergo LAAC with either device. The primary safety endpoint was a composite of procedure-related complications, all-cause mortality, or major bleeding at 12 months. The primary effectiveness endpoint was a composite of ischemic stroke or SE at 18 months, as well as the rate of LAA occlusion at 45 days, considering the dual occlusion mechanism (disc and lobe) for the Amulet device. The patients included in the study were at high risk for both stroke and bleeding, with a mean CHA_2_DS_2_-VASc score of 4.5 (Amulet) and 4.7 (Watchman), and a mean HAS-BLED score of 3.2 and 3.3, respectively. The results for the primary safety and effectiveness endpoints were similar between the two groups. However, device-related complications were more frequent in the Amulet group (4.5% vs. 2.5%), mainly due to pericardial effusion and device embolization. Following LAAC, patients in the Amulet group were prescribed aspirin plus clopidogrel or aspirin plus OAC (warfarin or a DOAC), while patients in the Watchman group received aspirin plus warfarin. If TEE at 45 days ruled out a peridevice leak ≥5 mm, then OAC was discontinued. After this evaluation, patients were advised to continue aspirin plus clopidogrel for up to 6 months, after which time clopidogrel was stopped and aspirin continued indefinitely. Device success, defined as effective LAA occlusion, was higher in the Amulet group (98.9%) compared with the Watchman group (96.8%), with more patients in the Amulet group achieving complete occlusion and no residual jet around the device.

The SWISS-APERO randomized clinical trial [31] was conducted in 221 patients and aimed to compare the Amulet and Watchman devices, with 77.3% of patients in the latter group receiving the Watchman FLX™ device. The primary endpoint was a composite of justified crossover to a non-randomized device during the LAAC procedure or residual LAA patency detected by cardiac computed tomography angiography (CCTA) at 45 days. The secondary endpoints included procedural complications, DRT, peridevice leak assessed by TEE, and clinical outcomes at 45 days. The Amulet device was not superior to the Watchman device regarding LAA patency at 45 days, which was high in both groups (67.6% vs. 70%, respectively), nor in the need for crossover to a non-randomized device. Major complications were higher in the Amulet group, mainly due to pericardial effusion and bleeding. The clinical outcomes were comparable between the two groups. The Watchman device was associated with a higher rate of peridevice leak as assessed by TEE.

The Amulet observational study [32] was a prospective, single-arm, non-randomized registry that included 1088 patients with AF, with mean CHA_2_DS_2_-VASc and HAS-BLED scores of 4.2 ± 1.6 and 3.3 ± 1.1, respectively. A total of 82.8% of the population had an absolute or relative contraindication to long-term anticoagulation, and 72.4% had experienced a previous major bleeding event. The primary aim of the study was to assess periprocedural and early clinical/TEE outcomes up to 3 months post-implantation. The device was implanted successfully in 99% of cases, with major adverse events occurring in 3.2% of patients during the procedure and index hospitalization. At discharge, patients were prescribed single antiplatelet therapy (23.0%), DAPT (54.3%), or OAC (18.9%). TEE evaluation, performed at a mean of 67 ± 23 days post-procedure in 673 patients, showed adequate occlusion (<3 mm leak) in 98.2% of cases, with DRT observed in 1.5% of patients.

The PINNACLE FLX trial [33] was a multicenter, non-randomized, prospective study designed to evaluate the safety and effectiveness of the new-generation Watchman FLX™ device in patients with AF for whom OAC was indicated. The primary safety endpoint was the occurrence of death, ischemic stroke, SE, or device- or procedure-related events requiring cardiac surgery within 7 days post-implantation. The primary effectiveness endpoint was successful LAAC, defined as a peridevice leak ≤5 mm as assessed by TEE at 12-month follow-up. A total of 400 patients were enrolled, with a mean CHA_2_DS_2_-VASc score of 4.2 ± 1.5 and a mean HAS-BLED score of 2.0 ± 1.0. Successful device implantation was achieved in 98.8% of cases. The incidence of the primary safety endpoint was 0.5%, meeting the predefined performance goal. Ten ischemic strokes occurred during the 1-year follow-up, with no cases of hemorrhagic stroke reported. The primary effectiveness endpoint was achieved in 100% of patients, and it also met the performance goal. No pericardial effusion occurred within the first 7 days. During the 1-year follow-up, four patients experienced pericardial effusion requiring intervention; however, none required cardiac surgery. There were no cases of device embolization, although seven patients developed DRT at the 1-year follow-up. After the procedure, patients were advised to take a DOAC (either apixaban or rivaroxaban) in combination with aspirin for up to 45 days, at which point a follow-up TEE was performed. If no peridevice leak >5 mm was detected, then the DOAC was discontinued, and DAPT with aspirin and clopidogrel was prescribed for 6 months, followed by aspirin monotherapy indefinitely. At 45 days, 96.2% of patients had adequate LAA exclusion and were able to discontinue DOAC therapy.

In the SURPASS observational study [34], 97,185 patients who underwent Watchman FLX™ implantation between 2020 and 2022 were analyzed. The key safety endpoint included all-cause mortality, ischemic stroke, SE, or device- or procedure-related complications requiring open cardiac surgery or major endovascular intervention from device implantation to hospital discharge. Successful implantation was achieved in 97.5% of patients, and the primary safety endpoint occurred in 0.45%. At the 1-year follow-up, all-cause mortality was 8.2%, and the stroke rate was 1.5%, of which 1.2% were ischemic strokes. Major bleeding was reported in 6.4% of patients. DRT was identified in 0.44% of patients based on TEE at 45 days. Regarding postprocedural antithrombotic therapy, the most common regimen was DOAC plus aspirin (47.5%), followed by DOAC monotherapy (24%). The study demonstrated outcomes comparable to those of the PINNACLE FLX trial in terms of implantation success and 1-year clinical results.

## 4. Ongoing and Planned Clinical Studies on Percutaneous Left Atrial Appendage Closure

The CHAMPION AF trial [35] is an ongoing study designed to compare LAAC as an alternative to DOAC therapy for stroke prevention in patients with AF for whom OAC is indicated. The trial plans to randomize up to 3000 patients with a CHA_2_DS_2_-VASc score ≥2 in men or ≥3 in women, in a 1:1 ratio, to receive either the Watchman FLX™ device or a DOAC. Patients in the LAAC arm are treated for at least 3 months post-implantation with either a DOAC plus aspirin, a DOAC alone, or DAPT, followed by aspirin or a P2Y12 inhibitor for up to 1 year. The control group receives DOAC therapy throughout the study. The total study duration is 5 years. Two primary endpoints will be assessed at 3 years: (1) a composite of stroke (ischemic or hemorrhagic), CV death, and SE to test for noninferiority, and (2) nonprocedural bleeding, tested for superiority in the LAAC group. A third co-primary endpoint—the noninferiority of the composite of ischemic stroke and SE—will be evaluated at 5 years (Table 2).

**Table 2.** **Ongoing studies on percutaneous left atrial appendage closure**.

Study	Device/comparator	Endpoints	Outcomes/results
CHAMPION AF (RT)	Watchman FLX/DOAC	Stroke, CV death and SE at 3 years (NI)	Ongoing
		Non-procedural bleeding at 3 years (superiority)	
		IS and SE at 5 years (NI)	
CATALYST (RT)	Amplatzer/DOAC	Composite of IS or SE; composite of IS, SE, or CV death (NI);	Ongoing
		Non-procedural major bleeding (S)	
CLOSURE-AF (RT)	Device/OAC	Stroke, SE, major bleeding, CV or unexplained death	Ongoing
OCCLUSION-AF	Device/DOAC	Stroke, SE, major bleeding, and all-cause mortality (NI)	Ongoing
STROKECLOSE (RT)	Amulet/MT	Stroke, SE, life threatening or major bleeding and all-cause mortality	Ongoing
ASAP-TOO (RT)	Watchamn/MT (single antiplatetelet therapy or no therapy)	- Effectiveness: IS, SE	Ongoing
		- Safety: all-cause death, IS, SE, device or procedure related event requiring cardiac surgery or major endovascular intervention	
CLEARANCE (RT)	LAAC/DOAC	Stroke, SE, severe bleeding, or CV/unexplained death	Ongoing
COMPARE LAAO (RT)	LAAC/MT (single/DAPT or no therapy)	- Efficacy: stroke, TIA or SE	Ongoing
		- Safety: 30-day peri-procedural complications	
LAA-KIDNEY (RT)	LAAC/MT (OAC or antiplatelet therapy)	Composite of stroke, SE, CV or unexplained death and major bleeding	Ongoing

CV, cardiovascular; DOAC, direct oral anticoagulation; IS, ischemic stroke; LAAC, left atrial appendage closure; MT, medical therapy; NI, non-inferiority; OAC, oral anticoagulation; RT, randomized trial; S, superiority; DAPT, dual antiplatelet therapy.

Other ongoing studies include:

- CATALYST [36] compares LAAC using the AMPLATZER Amulet device with DOAC therapy.
- CLOSURE-AF [37], compares LAAC with either DOACs or VKAs in patients at high risk of stroke and bleeding.
- OCCLUSION-AF [38] trial aims to determine whether LAAC is non-inferior to DOACs in reducing a combined endpoint of stroke, SE, major bleeding, and all-cause mortality in patients with AF who recently experienced an ischemic stroke or transient ischemic attack (TIA).
- STROKECLOSE [39] evaluates LAAC versus medical therapy (OAC, antiplatelet therapy, or no antithrombotic therapy) in patients with a history of intracranial hemorrhage.
- ASAP-TOO [40] compares LAAC with the Watchman device against medical therapy (single antiplatelet therapy or no therapy) in patients unsuitable for OAC.
- CLEARANCE [41] compares LAAC with DOAC in patients with a prior episode of intracranial bleeding (>6 weeks before enrollment).
- COMPARE-LAAO [42] is a randomized trial designed to evaluate the safety and efficacy of LAAC in patients with AF who are ineligible for OAC. Patients in the device group will receive DAPT, while those in the control group will be managed with either no antithrombotic therapy or single antiplatelet or DAPT therapy, at the investigator’s discretion.
- LAA-KIDNEY [43] is a randomized trial designed to compare LAAC using the Amplatzer Cardiac Plug and/or Amulet device with medical therapy in patients with AF at high risk of both stroke and bleeding and with end-stage renal disease (ESRD).

## 5. Imaging Modalities for Evaluation and Planning of Left Atrial Appendage Closure

Once LAAC is indicated, the LAA must be carefully evaluated anatomically and to exclude the presence of thrombus. TEE and CCT are the two principal imaging modalities for LAA assessment. Both provide accurate measurements, although CCT tends to yield slightly larger dimensions and predicts the optimal device size more reliably [44, 45]. Moreover, the anatomical ostium of the LAA is not always circular and may present various shapes—such as oval (the most common, 68.9% of cases), foot like, triangular, water drop like, and round—so the use of the perimeter-derived diameter, assessed by CCT, is the most reliable parameter to predict the optimal LAA occluder size [18]. In addition, CCT demonstrates high diagnostic accuracy for thrombus detection, particularly when delayed contrast protocols are used, making it a valuable alternative to TEE [46].

Three-dimensional (3D) TEE has become an essential tool in the preprocedural evaluation of LAAC. It provides high sensitivity for differentiating thrombus from pectinate muscles within the LAA, an important distinction for patient selection and procedural safety. Moreover, it enables detailed assessment of the spatial relationships between the LAA and the surrounding cardiac structures, thereby enhancing procedural planning [17].

Proper device sizing is critical to ensure stability and to complete sealing of the LAA. Undersizing increases the risk of device embolization, whereas oversizing may cause LAA perforation and/or pericardial effusion [47, 48]. Ideally, measurements for device selection should be obtained at end systole, when the LAA reaches its maximum dimension, and with adequate intravascular volume to avoid underestimation of size. The ostium, landing zone, and depth measurements should be obtained from multiple TEE views, both pre- and intraprocedurally. The LAA ostium is most often oval, and its largest orifice diameter is best visualized in the 120°–135° imaging planes [49]. Real-time 3D TEE provides more accurate measurements of the LAA orifice, correlating better with CCT-based measurements, while 2D TEE tends to underestimate the orifice area [50, 51].

As mentioned earlier, LAA classification based solely on morphological variants does not adequately predict procedural difficulty due to the structure’s inherent complexity. Therefore, additional anatomical and functional factors should be considered during preprocedural planning.

**Fig. 4.**
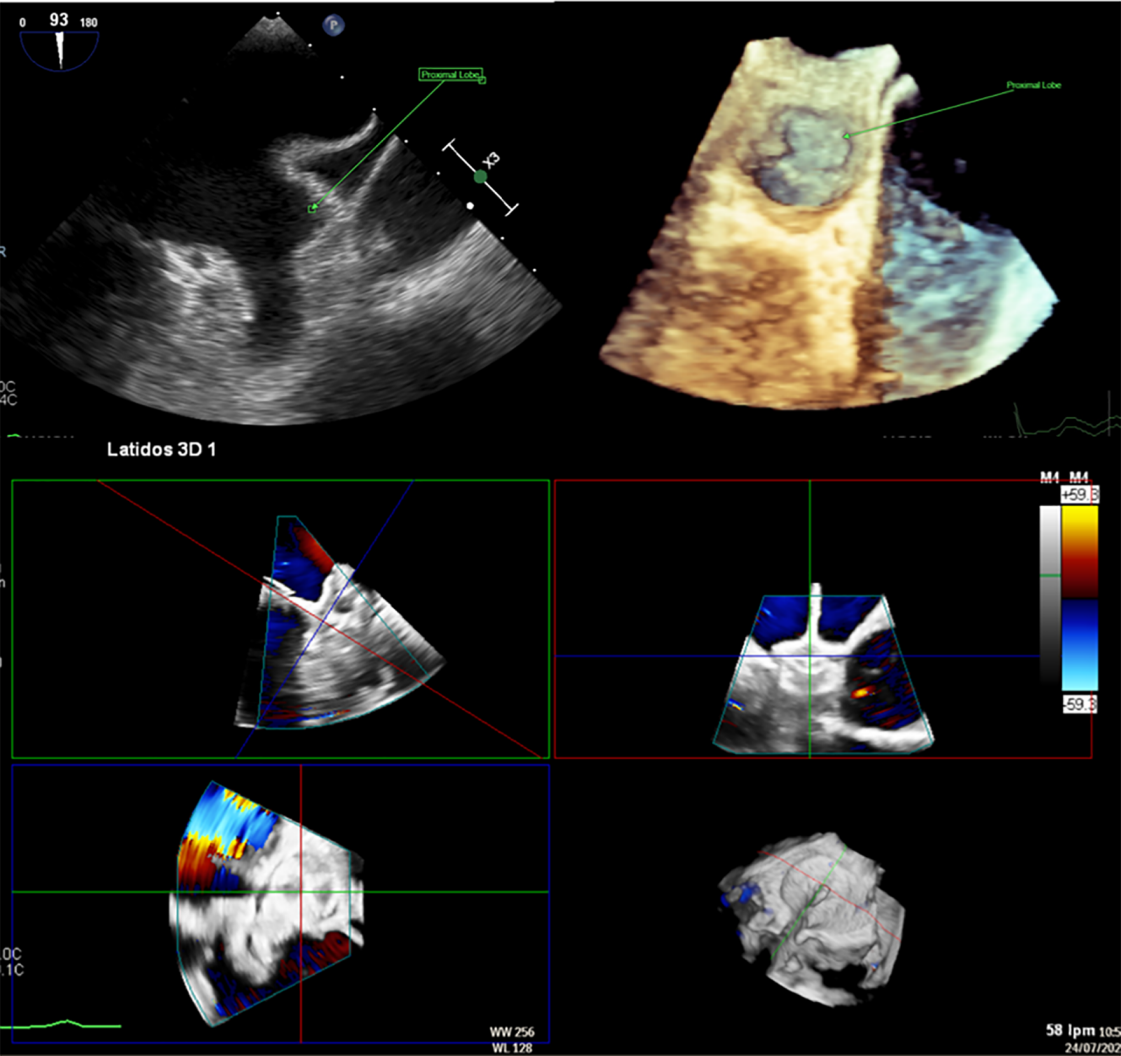
**Left atrial appendage closure (LAAC) in a patient with complex anatomy**. Transesophageal echocardiography (TEE) revealed a left atrial appendage (LAA) with a large proximal lobe, presenting a challenge for percutaneous closure. Nevertheless, an Amulet device was deployed successfully, with the lobe of the device positioned in the proximal lobe of the LAA and the disc effectively sealing the anatomical ostium.

The European Left Atrial Appendage Closure Club (ELAACC) recently proposed the ELAAC classification system [52], which evaluates five key anatomical and functional domains: entrance/ostium, landing zone, overall anatomy, axis/orientation, and contractility of the LAA. Each domain encompasses features that can influence procedural planning and outcomes. For example, a large ostium or landing zone (>30 mm), an eccentric landing zone (defined as Ømax/Ømin >1.5), or the presence of a proximal lobe—an outpouching >10 mm in both width and depth at the level of the landing zone—may increase procedural complexity and impact success rates. Based on the presence or absence of 12 key parameters, the authors proposed the ELAACC score, which categorizes LAA anatomy into three levels of complexity: *simple* (0 complex features), *moderate* (1–2 features), and *challenging* (≥3 features). In their initial experience, 21.5% of cases were classified as challenging. Although the ELAACC score is based on expert consensus and requires further validation, it represents a useful tool for interventionalists to enhance preprocedural planning and minimize the risks associated with complex anatomical and functional LAA variants (Fig. 4).

## 6. Evidence-Based Indications for Percutaneous Left Atrial Appendage Closure

Over the past two decades, there have been significant advances in LAAC regarding both device technology and operator expertise, resulting in a safer and more effective procedure. As evidence has accumulated, LAAC has emerged as an important option to prevent stroke in patients with AF. In the 2023 American College of Cardiology/American Heart Association/American College of Clinical Pharmacy/Heart Rhythm Society (ACC/AHA/ACCP/HRS) AF guidelines, the indication for LAAC has been upgraded from Class IIb to Class IIa for patients with a moderate-to-high risk of stroke who have a contraindication to long-term OAC therapy [53]. Conversely, the 2024 European guidelines [1] adopt a more conservative approach, assigning LAAC a Class IIb, Level of Evidence C recommendation, indicating that it may be considered in patients with a contraindication to long-term OAC.

Beyond patients with a formal contraindication to anticoagulation, several additional clinical settings have emerged where LAAC may confer benefit, and its use is expanding accordingly. While DOACs are safer and more effective than VKAs for stroke prevention, there is a residual risk of recurrent stroke or SE among patients treated with DOACs, ranging from 2.0 to 2.8 per 100 patient-years [54, 55, 56]. The subgroup that experiences recurrent cerebrovascular events despite adequate anticoagulation poses a significant clinical challenge. Evidence suggests that LAAC in such patients may reduce the incidence of stroke compared with the risk predicted by their CHA_2_DS_2_-VASc score [57].

Aarnink *et al*. [58] compared the outcomes of patients who experienced thrombotic events despite OAC with those who had contraindications to OAC and found comparable stroke rates. Therefore, LAAC is emerging as a potential alternative for patients without a formal contraindication to OAC but with recurrent stroke or SE, offering an additional percutaneous option for stroke prevention.

Chronic kidney disease (CKD) is closely associated with AF, with a markedly higher prevalence among patients with impaired renal function. Across all CHA_2_DS_2_-VASc strata, patients receiving renal replacement therapy face an elevated risk of stroke or SE [59], while also being predisposed to major bleeding—especially under OAC therapy.

The use of DOACs in advanced CKD remains controversial, particularly in Europe. In the United States, however, the use of DOACs, especially apixaban, has increased among patients with AF and ESRD, owing to its relatively lower renal clearance. In a large retrospective analysis, Siontis *et al*. [60] demonstrated that apixaban was associated with a lower rate of major bleeding than warfarin in patients with ESRD and AF undergoing dialysis, and it maintained comparable efficacy in the prevention of stroke and SE. Nevertheless, the rate of major bleeding in that study was 19.7 events per 100 person-years, nearly ninefold higher than in the ARISTOTLE trial, where patients with severe renal impairment (serum creatinine >2.5 mg/dL or creatinine clearance <25 mL/min) were excluded. Similarly, the RENAL-AF trial [61], which compared apixaban with warfarin in patients receiving hemodialysis, reported a high incidence of major or clinically relevant non-major bleeding (32% vs. 26%, respectively) at the 1-year follow-up. In this context, LAAC offers a promising alternative to OAC in patients with both AF and CKD, as it has been associated with reduced annual rates of thromboembolic and bleeding events [62].

Catheter ablation represents another interventional option for rhythm control in patients with symptomatic AF. It is recommended for those with paroxysmal or persistent AF who are resistant or intolerant to antiarrhythmic drugs. However, continuation of OAC after ablation is still advised for patients at elevated thromboembolic risk—regardless of the rhythm outcome—to prevent ischemic stroke [1]. The Option trial [63] was a randomized study enrolling patients with AF with a moderate-to-high risk of stroke who underwent catheter ablation. It compared concomitant LAAC with continued OAC therapy. In the LAAC arm, patients received OAC plus aspirin for 90 days, followed by aspirin monotherapy for up to 12 months. The primary safety endpoint—non-procedure-related major or clinically relevant non-major bleeding—was significantly reduced in the LAAC group at 36 months. The primary efficacy endpoint, a composite of all-cause death, stroke, or SE, met the criterion for noninferiority. Notably, 95% of participants received a DOAC, and most had a low-to-moderate bleeding risk. Non-procedure-related bleeding occurred in 18.1% of patients in the OAC group versus 8.5% in the device group, mainly driven by clinically relevant non-major bleeding.

Combining LAAC with AF ablation can be advantageous, as both procedures share similar technical steps—including femoral venous access, transseptal puncture (TSP), and fluoroscopic guidance—potentially reducing the number of hospitalizations. However, this combined strategy also carries procedural risks, such as an increased duration, greater exposure to radiation, and the need for tight coordination between the electrophysiology and interventional teams. Moreover, there are limited long-term data, and additional studies are required to fully establish the safety and efficacy of this combined approach.

Finally, it is important to evaluate an effective and cost-efficient analysis of LAAC in order to improve patient selection and identify risk factors associated with early mortality after the procedure. Aarnink *et al*. [64] analyzed data from the EWOLUTION registry and reported a 16.4% mortality rate at 2 years, with 50% of deaths due to non-cardiovascular causes. The authors identified six independent predictors of mortality, including age, heart failure, valvular disease, vascular disease, and abnormal liver and renal function.

## 7. Technical and Procedural Guide to Percutaneous Left Atrial Appendage Closure

This section provides a detailed, step-by-step description of the percutaneous LAAC procedure. It can be performed under either general anesthesia or conscious sedation. Although intracardiac echocardiography (ICE) can be used for procedural guidance, TEE remains the standard imaging modality in most catheterization laboratories. A micro-TEE probe, which offers high-resolution imaging, can be used as an alternative to the standard probe and allows the procedure to be performed without general anesthesia. In our practice, patients undergo preprocedural TEE to assess their tolerance. If they demonstrate adequate cooperation, then we proceed with LAAC using micro-TEE under conscious sedation. We recommend that femoral venous access be obtained under ultrasound guidance to minimize the risk of inadvertent arterial puncture and to reduce vascular complications. In our practice, we administer 2000 units of unfractionated heparin following venous puncture. Radial arterial access is typically required for continuous pressure monitoring during the procedure. A pigtail catheter may be positioned by radial approach in the ascending aortic root to facilitate the TSP, although this step is often unnecessary when adequate TEE guidance is available.

**Fig. 5.**
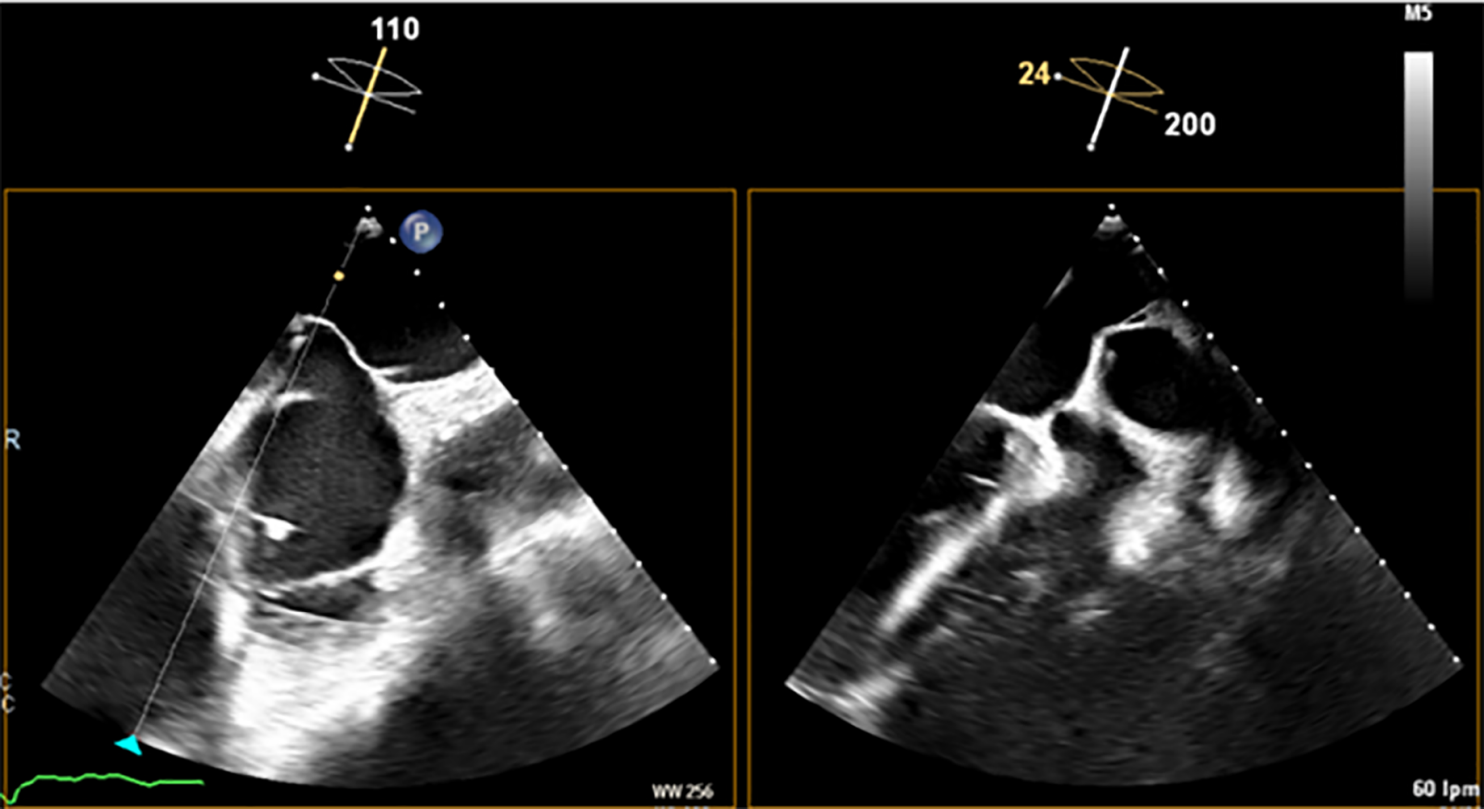
**Transesophageal echocardiography (TEE) shows the tenting effect of the transseptal needle on the fossa ovalis in an inferior (left) and posterior (right) position**.

**Fig. 6.**
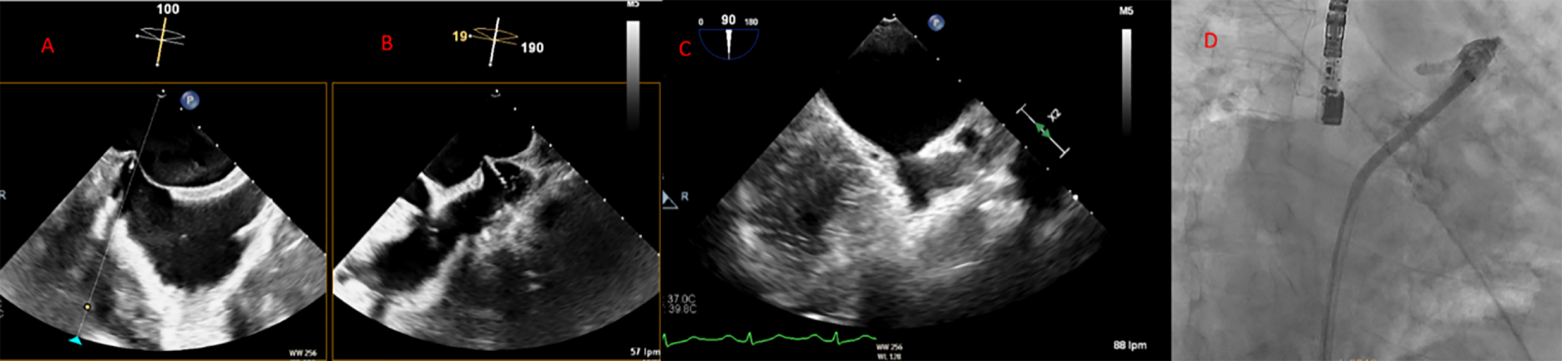
**Middle and inferior transseptal puncture (TSP) approach**. A transseptal puncture (TSP) in the inferior (A) and middle (B) portions of the fossa ovalis in a patient with an inverse chicken-wing morphology (C) of the left atrial appendage (LAA), clearly visualized in the angiographic image (D).

**Fig. 7.**
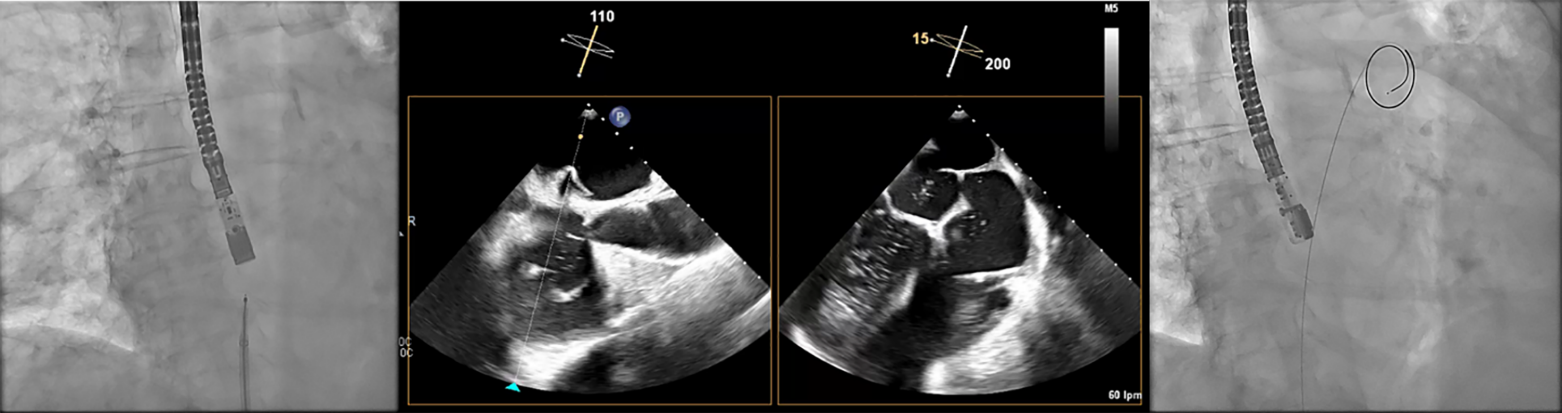
**Transseptal puncture (TSP) using the VersaCross system**. The Versacross wire, within its sheath, is seen in contact with the interatrial septum under fluoroscopy (left). Transesophageal echocardiography (TEE) reveals tenting at the level of fossa ovalis and the wire crossing into the left atrium (middle), where it immediately assumes a pigtail shape (right), providing a safe means of crossing the septum.

One of the most critical steps in LAAC is the TSP. The LAA is typically located in the anterosuperior portion of the LA and directed laterally and anteriorly. Consequently, an inferoposterior puncture within the fossa ovalis provides the most favorable trajectory for aligning the access sheath along the LAA’s long axis, thereby facilitating precise device positioning (Fig. 5) [15]. In cases where the LAA includes an additional, very anteriorly oriented lobe, a mid-fossa puncture—rather than an excessively posterior one—may provide better alignment with the target segment (Fig. 6). A TSP is traditionally performed using a Brockenbrough needle in combination with a transseptal sheath (Abbott, Abbott Park, IL, USA). We prefer the VersaCross® Transseptal System (Baylis Medical, part of Boston Scientific Corporation, Marlborough, MA, USA), which integrates a radiofrequency (RF) puncture system with an exchange-ready pigtail wire. This design facilitates both safe LA access and efficient navigation during the procedure. The pigtail wire, positioned within the transseptal sheath, is connected to an RF generator. Once the sheath tents the fossa ovalis appropriately, RF energy is applied, allowing the guidewire to cross into the LA, where it safely coils, thereby minimizing the risk of atrial wall injury (Fig. 7).

**Fig. 8.**
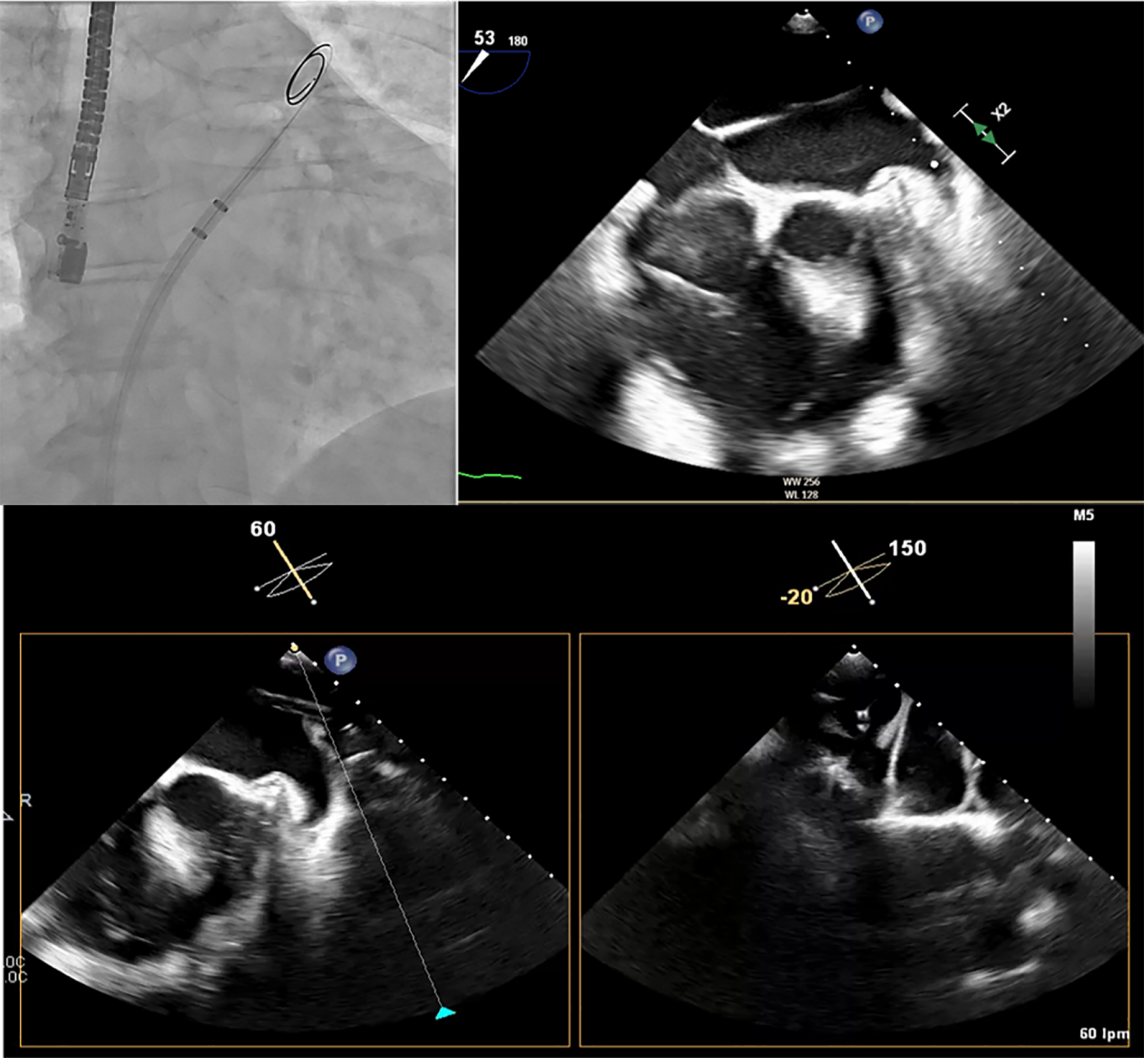
**Advancement of the sheath over the VersaCross wire**. The Versacross wire is positioned in the left superior pulmonary vein (LSPV), as seen in the fluoroscopy image (upper left) and confirmed by transesophageal echocardiography (TEE) (upper right). The dedicated sheath for left atrial appendage closure (LAAC) device was advanced successfully over the same wire up to the LSPV, as visualized by TEE (bottom image).

Immediately after a successful TSP, additional unfractionated heparin is given to reach a total dose of 100 U/kg. Then, the pigtail-shaped guidewire, which offers high support, is advanced and coiled within the LSPV by gentle clockwise rotation. This position allows smooth advancement of the VersaCross sheath into the LA for septal dilation. We maintain the same wire in the LSPV for the subsequent advancement of the dedicated LAAC delivery sheath (Fig. 8). Once the dedicated sheath reaches the LA and is positioned near the LSPV, a pigtail catheter is advanced over the VersaCross wire into the LA, after which the wire is withdrawn. With a gentle counterclockwise rotation of the assembly, the pigtail is directed and positioned within the LAA. At this stage, LAA angiography is performed in two standard projections: right anterior oblique (RAO) 30°/cranial 20° and RAO 30°/caudal 20°, which correspond to the 45° and 135° TEE views, respectively (Table 3). Guided primarily by TEE—and secondarily by angiography—the device size is selected following precise evaluation of the landing zone and theoretical device positioning within the LAA. Although several occlusion devices are currently available (Table 4), we provide a detailed procedural overview of two of the most widely used LAAC systems worldwide.

**Table 3.** **Correlation of standard TEE and fluoroscopic imaging views**.

TEE view	Fluoroscopic view
0°	AP
45°	RAO 30°, Cranial 20°
90°	RAO 30°
135°	RAO 30°, Caudal 20°

TEE, transesophageal echocardiography; AP, antero-posterior; RAO, right anterior oblique.

**Table 4.** **Left atrial appendage occlusion devices (CE-mark)**.

Device	Manufacturer	Features	CE mark
Amplatzer Amulet	Abbott Vascular (USA)	16–34 mm (8 sizes)	2013
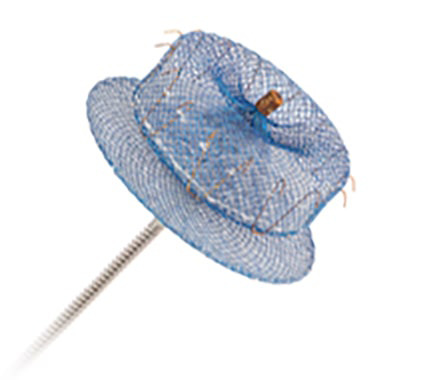		Sheath: 12 and 14 F	
Watchman FLX	Boston Scientific (USA)	20–35 mm (5 sizes)	2019
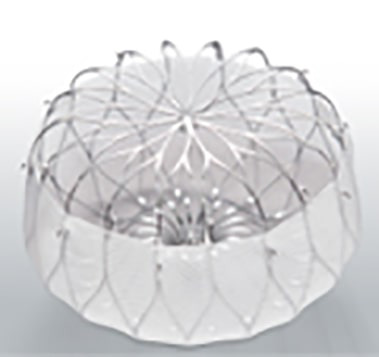		Sheath: 14 F	
WaveCrest	Biosense Webster (USA)	22, 27 and 32 mm	2013
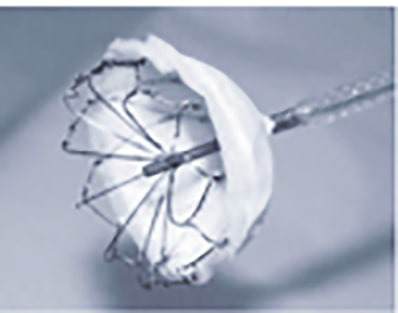		Sheath: 15 F	
Occlutech	Occlutech (Sweden)	18–33 mm (6 sizes)	2016
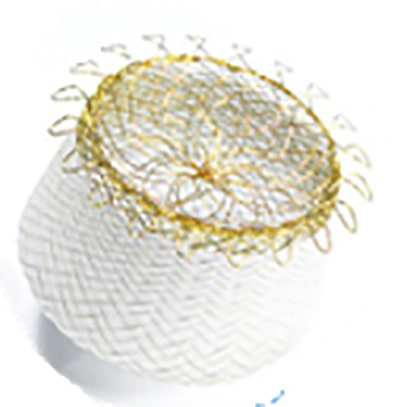		Sheath: 12 and 14 F	
LAmbre	Lifetech Scientific (China)	16–36 mm (17 sizes)	2016
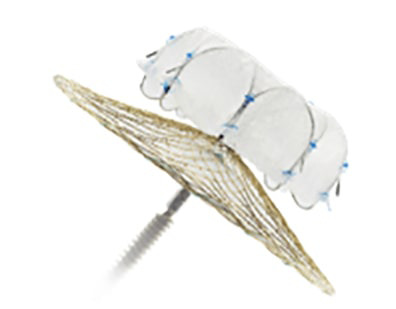		Sheath: 8, 9, and 10 F	
Ultraseal	Cardia, Inc. (USA)	16–34 mm (10 sizes)	2016
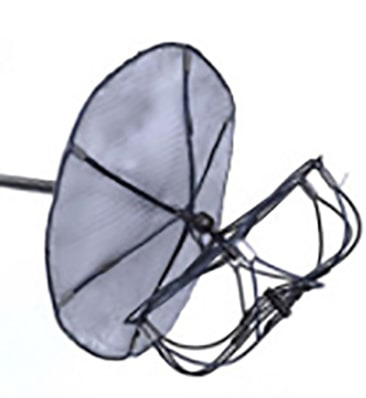		Sheath: 9–12 F	

**CE**: European Union conformity marking.

### 7.1 Watchman FLX™: Technical Features and Procedural Considerations

The new-generation Watchman FLX™ device (Boston Scientific Corporation) represents an advanced evolution of the original Watchman LAAC system. The most recent iteration, the WATCHMAN FLX™ Pro, introduces three notable innovations:

1. HEMOCOAT™ technology, designed to enhance endothelialization, reduce platelet adhesion, and promote more complete device healing;
2. Enhanced radiopaque markers for improved fluoroscopic visibility and precise positioning; and
3. A new 40-mm device size, accommodating larger appendages.

During the preparation of this article, the WATCHMAN FLX™ Pro had limited availability in Europe. Therefore, the following description refers to the Watchman FLX™ model currently in use in our laboratory and reflects our own procedural experience.

#### 7.1.1 Device Design

**Fig. 9.**
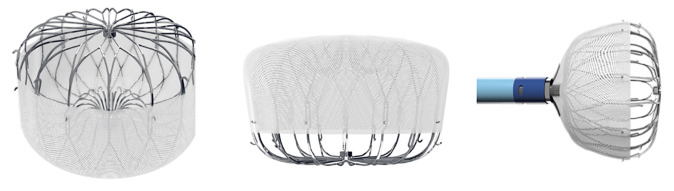
**The Watchman FLX™ device has two rows of 9 J-shaped anchors, with a total of 18 anchors (reproduced with permission from Boston Scientific)**.

The Watchman FLX™ features a self-expanding nitinol frame with two rows of nine J-shaped fixation anchors (18 in total, compared with 10 in the previous generation) (Fig. 9). The atrial-facing surface is covered by a permeable polyester fabric, and the closed, atraumatic distal end minimizes the risk of LAA perforation. These structural refinements improve device conformability, enhance anchoring stability, reduce peridevice leaks, and enable full or partial recapture and repositioning. The device is available in five sizes, covering landing zone diameters from 14 to 31.5 mm (Table 5).

**Table 5.** **Device sizing is based on the diameter of the landing zone (reproduced with the permission of Boston Scientific)**.

Max LAA Ostium Width and/or Deployed Closure Device Diameter (mm)	Closure Device Size (mm)
14.0–18.0	20
16.8–21.6	24
18.9–24.3	27
21.7–27.9	31
24.5–31.5	35

#### 7.1.2 Device Delivery System and Preparation

**Fig. 10.**
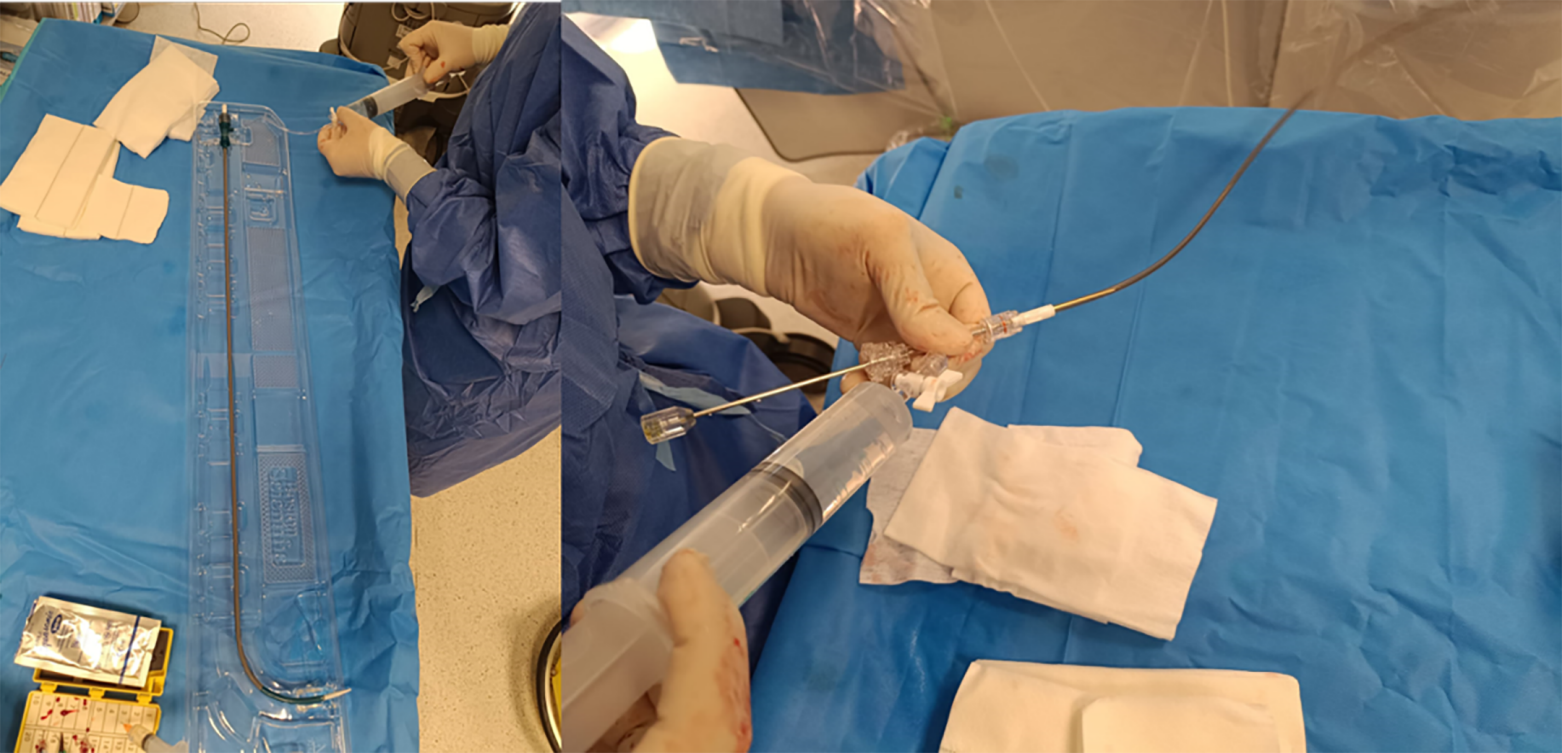
**The Watchman FLX™ sheath (left) alongside its delivery system containing the preloaded device (right)**.

The Watchman FLX™ is delivered via a dedicated sheath and delivery system with the device preloaded (Fig. 10). After advancing the sheath toward the LAA—keeping the pigtail catheter ahead for safety—angiographic visualization of the LAA is performed.

**Fig. 11.**
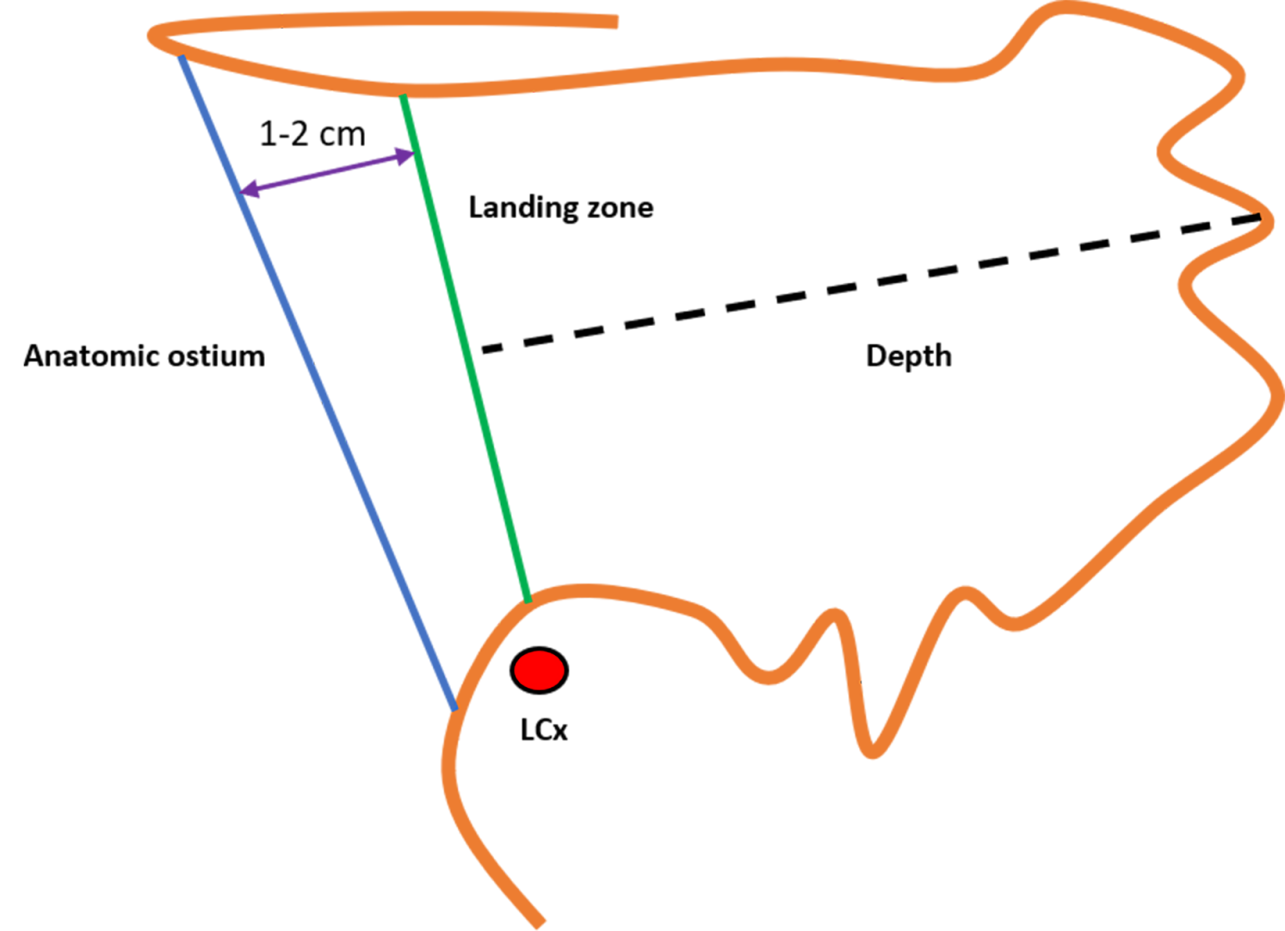
**Measurement of the landing zone and depth of the left atrial appendage (LAA) for the Watchman FLX™ device**. LCx, left circumflex artery.

**Fig. 12.**
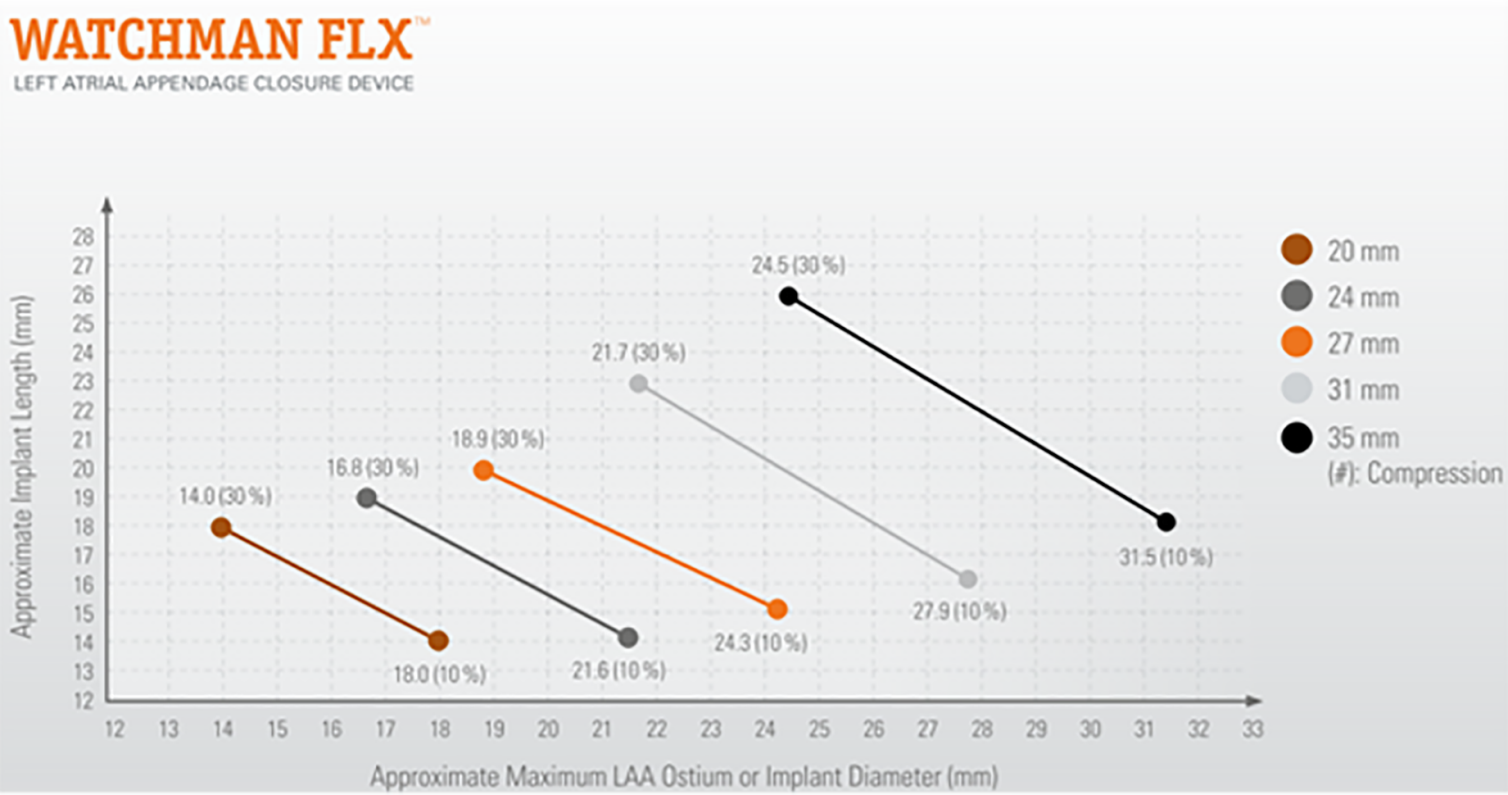
**Watchman FLX™ size selection is based on the diameter of the left atrial appendage (LAA) landing zone**. The recommended compression range is 10%–30%. The required depth for device implantation may vary depending on the degree of compression (reproduced with permission from Boston Scientific).

Because the Watchman FLX™ functions as an occlusive device, precise measurement of the landing zone and depth is critical for appropriate sizing. The landing zone is defined by a line drawn from the inferior aspect of the LAA at the level of the LCx to a point 1–2 cm distal to the ridge adjacent to the LSPV. The LAA depth is then measured perpendicularly from this line (Fig. 11) [17]. Device size selection follows the manufacturer’s recommended sizing and compression criteria (Fig. 12).

**Fig. 13.**
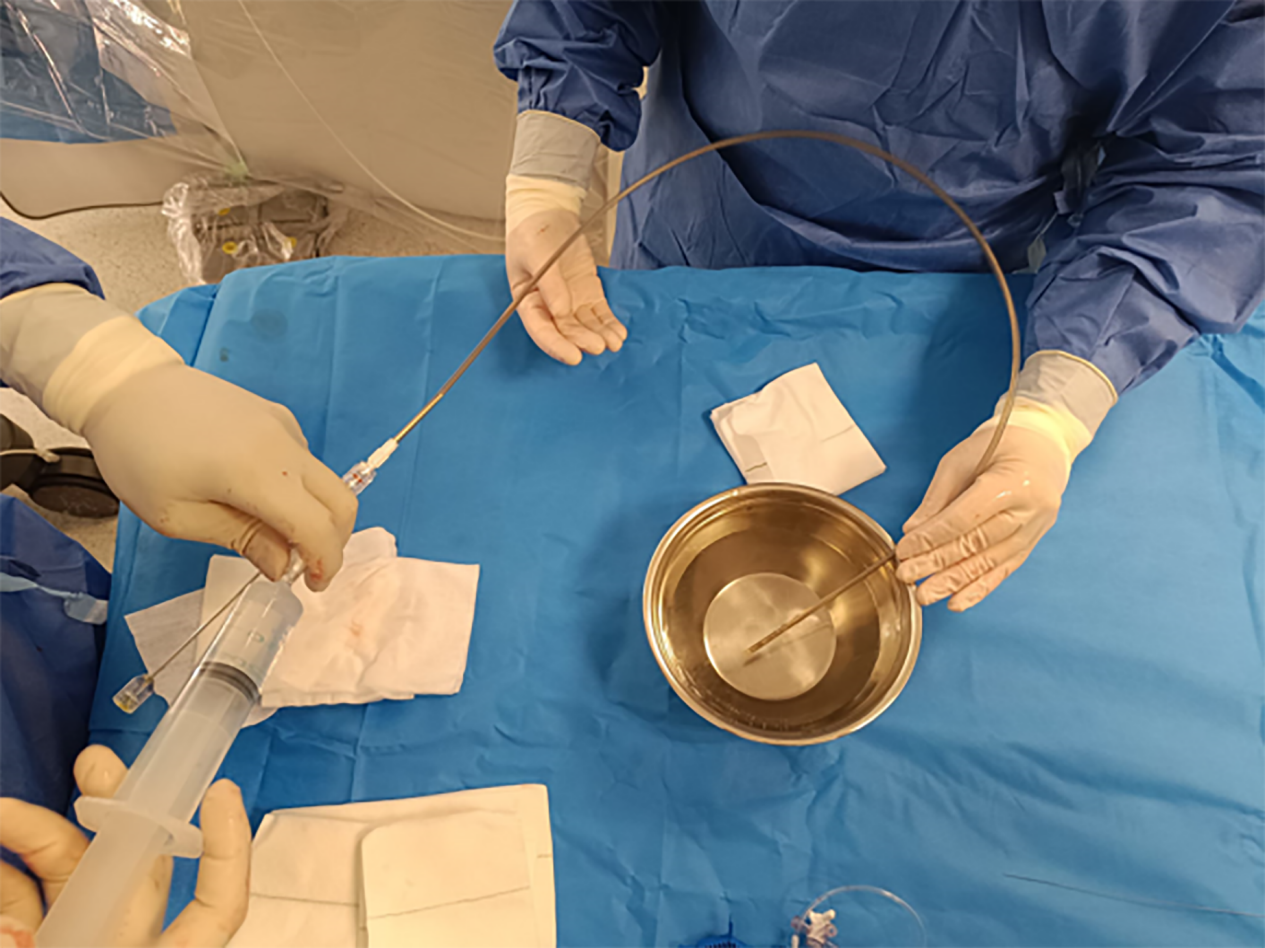
**The delivery system, which includes the preloaded device, should be meticulously flushed before being introduced into the sheath**.

Before use, the delivery system must be thoroughly flushed to prevent air embolism (Fig. 13). Proper alignment between the device and the distal radiopaque marker of the delivery catheter should be verified. If misalignment is observed, then minor advancement or retraction of the deployment handle can correct it.

#### 7.1.3 Device Deployment

**Fig. 14.**
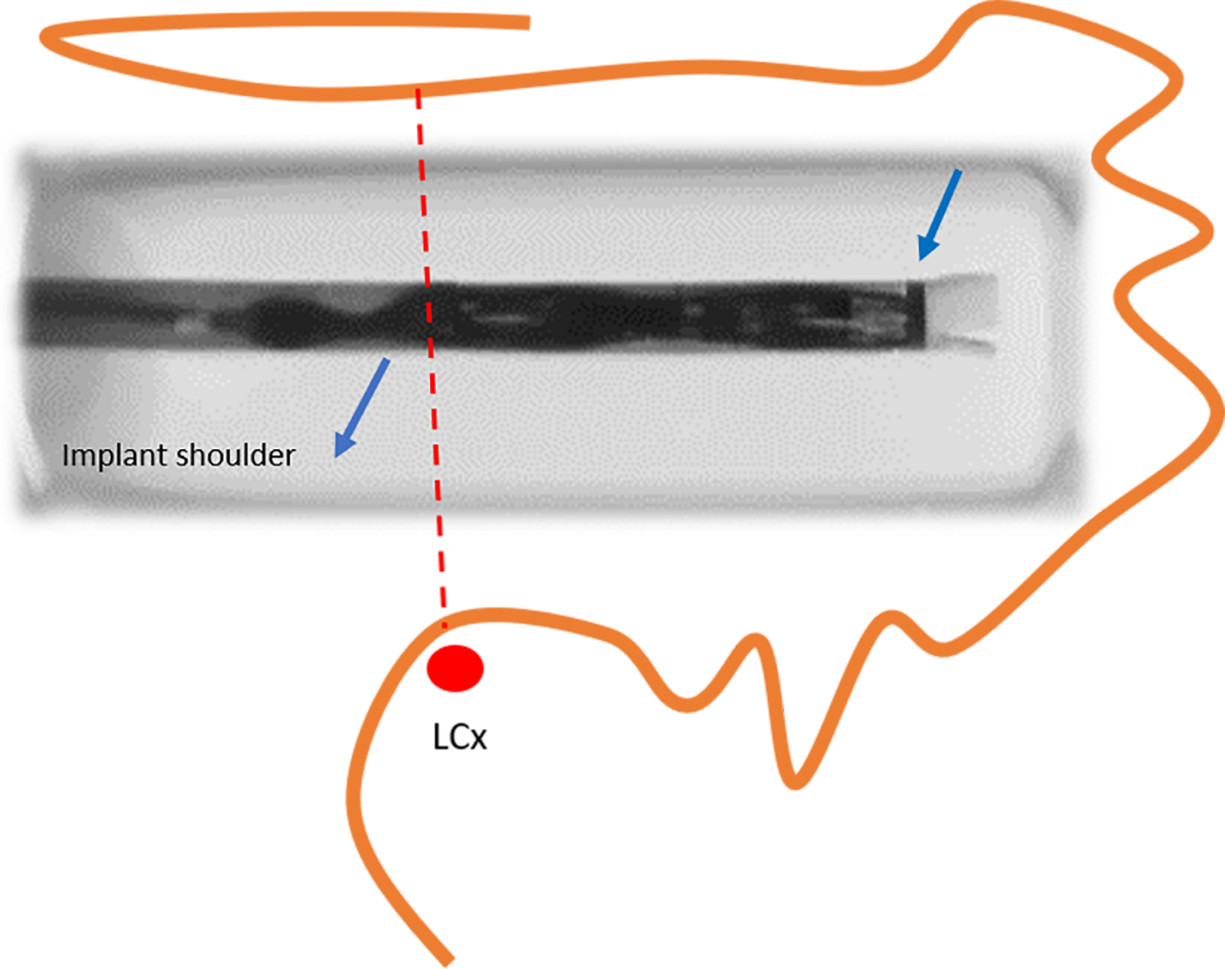
**Alignment of the distal tip of the device with the distal marker of the sheath (right arrow)**. At this stage, the device shoulder should ideally be positioned at the level of the left circumflex artery (LCx) (reproduced with permission from Boston Scientific).

**Fig. 15.**
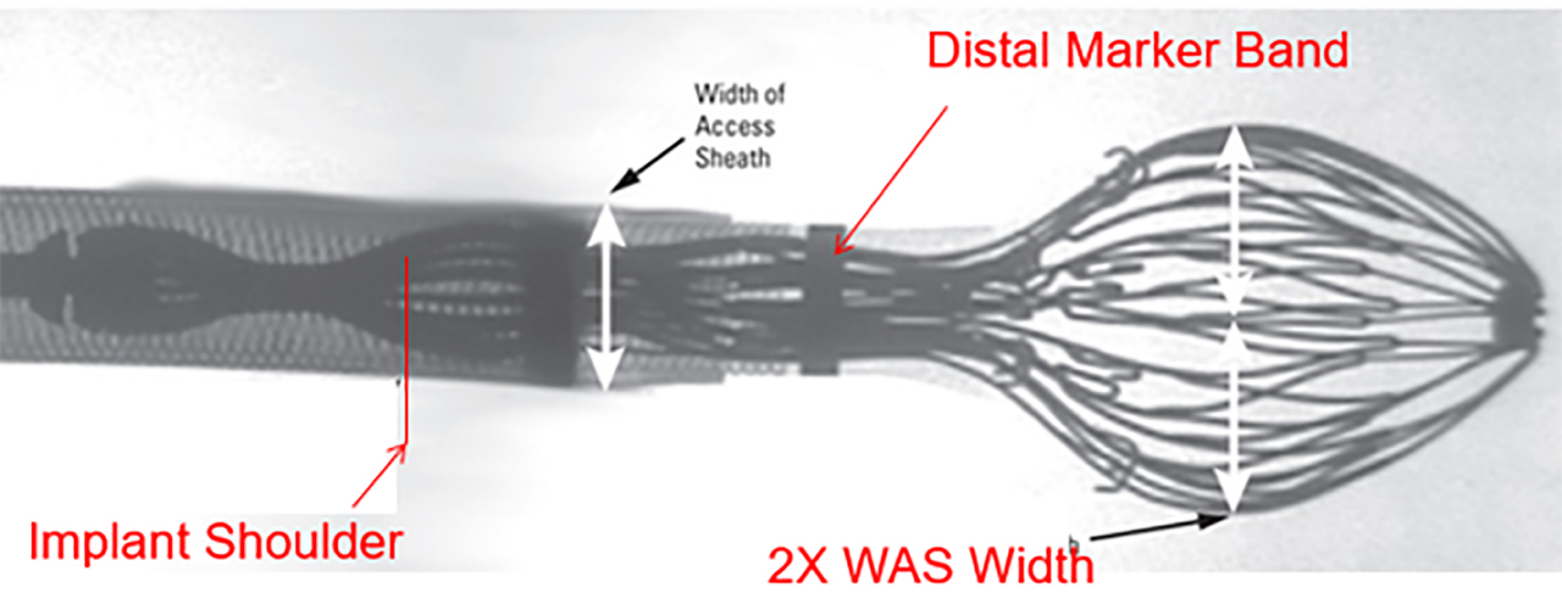
**While the delivery handle is held fixed, the sheath and the delivery system are pulled back to form a “ball” configuration approximately twice the diameter of the sheath (reproduced with permission from Boston Scientific)**.

While maintaining continuous flushing, the delivery system is advanced into the sheath until the distal tip of the device aligns with the distal marker of the sheath already positioned within the LAA (Fig. 14). With the delivery system held steady, the sheath is retracted until both components are fully connected. Then, keeping the deployment knob fixed, the sheath and delivery system are simultaneously withdrawn to create a “ball” configuration, approximately twice the diameter of the access sheath (Fig. 15). In this configuration, the entire system (i.e., the access sheath and the delivery system) can be safely advanced or retracted to fine-tune the alignment of the device shoulder with the landing zone. This configuration is a major improvement over the first-generation Watchman™: In the “ball” position, the distal anchors remain unexposed, allowing safe advancement or retraction of the system without risk of tissue injury. Once the shoulder of the device aligns with the landing zone, the sheath and delivery catheter assembly are retracted relative to the deployment knob to release the device. Slight forward pressure should then be applied to ensure full expansion and proper sealing of the device.

#### 7.1.4 TEE-Guided Assessment of Closure

**Fig. 16.**
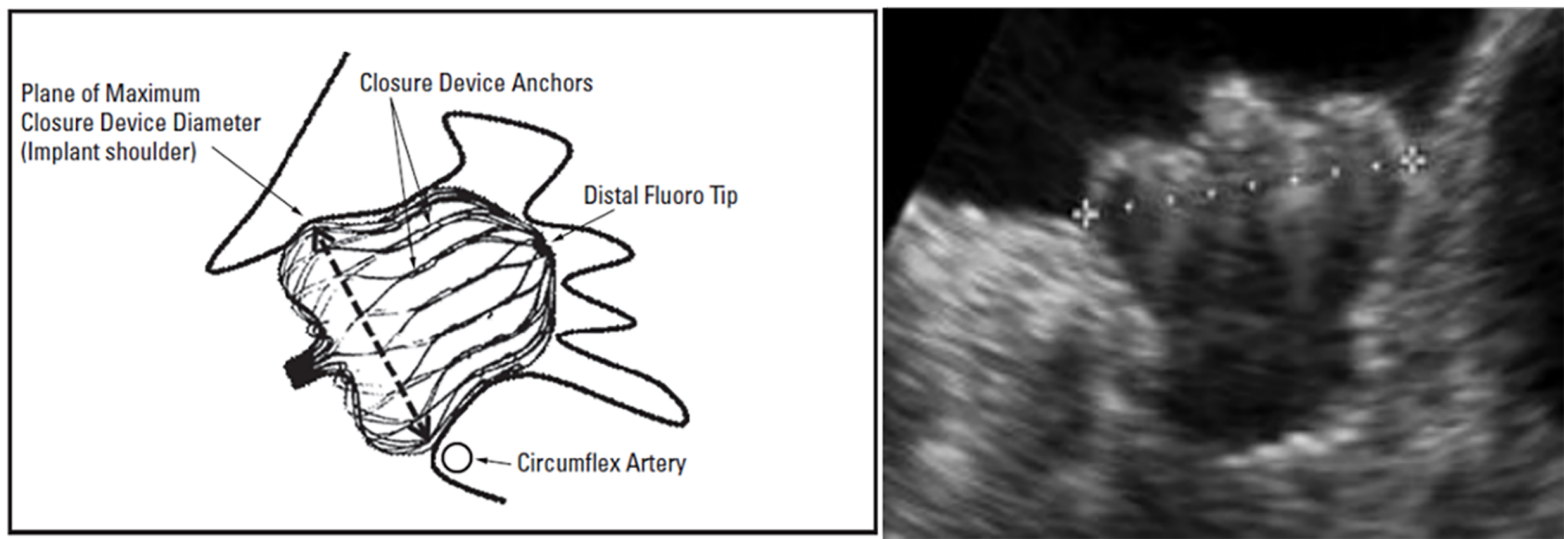
**The shoulder of the Watchman FLX™ device positioned at the level of the left circumflex artery (LCx) (reproduced with permission from Boston Scientific)**.

Following deployment, TEE guidance is essential to confirm correct positioning and function:

- The maximum device diameter should be located at or just distal to the landing zone (Fig. 16).
- A gentle tug test confirms appropriate anchoring and stability.
- Device compression at its widest point should fall within 10%–30%.
- Sealing should be complete, with no leak or residual peridevice flow <5 mm.

#### 7.1.5 Device Repositioning and Final Release

If repositioning is required, then the Watchman FLX™ can be partially or fully recaptured to disengage the anchors from the LAA wall. The “ball” configuration can then be recreated, and deployment repeated. Once satisfactory positioning and sealing are confirmed, final release is performed by advancing the sheath as close as possible to the device while applying counterclockwise rotation to the deployment knob.

#### 7.1.6 Vascular Closure

**Fig. 17.**
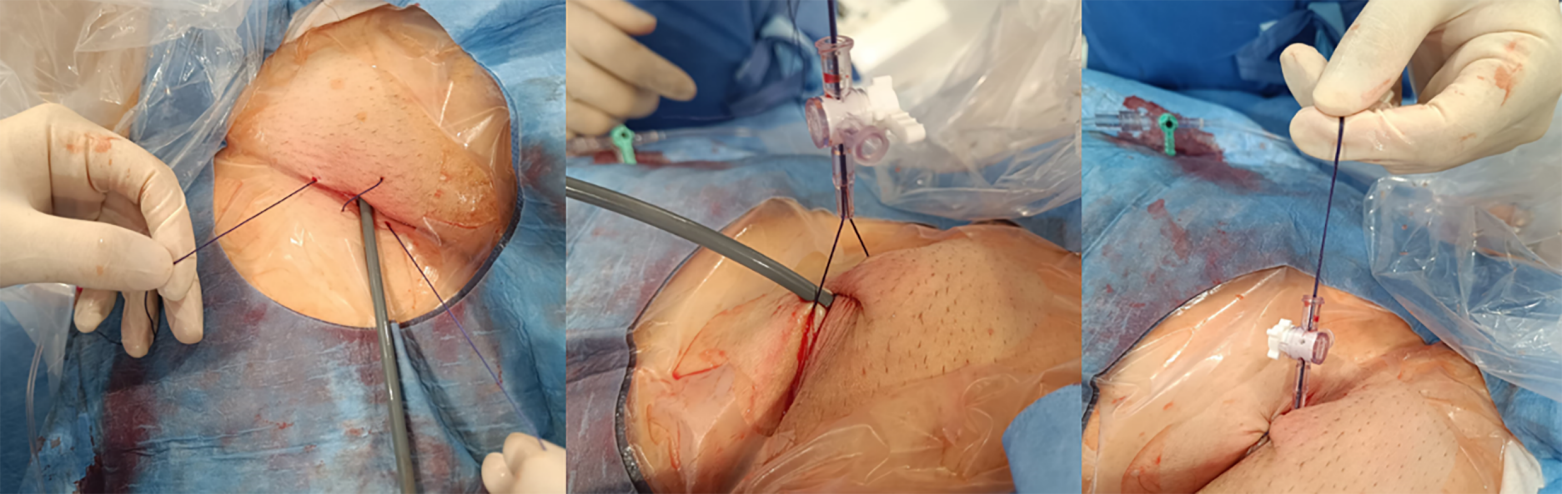
**Figure-of-eight suture using the side port of an introducer sheath**.

For femoral access site management, we routinely use a figure-of-eight suture technique. The sutures are passed through the side port of the introducer (previously cut), tension is applied to achieve hemostasis, and the closure is secured by locking the stopcock (Fig. 17).

### 7.2 Amplatzer Amulet: Technical Features and Procedural Considerations

**Fig. 18.**
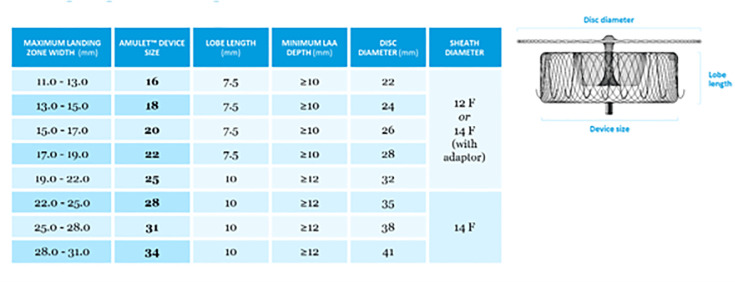
**Amulet device sizing according to the landing zone measurements and the minimum depth required for deployment (reproduced with permission from Abbott)**.

The Amplatzer Amulet device (Abbott, St. Paul, MN, USA) is another system for LAAC that is widely used in contemporary practice. It consists of a distal lobe and a proximal disc, designed to achieve complete exclusion of the LAA from systemic circulation. The device is available in eight sizes, covering a landing zone diameter ranging from 11 to 31 mm (Fig. 18).

#### 7.2.1 Device Design and Mechanism of Occlusion

The Amulet achieves LAA exclusion through two complementary mechanisms:

1. The lobe, which anchors the device within the appendage and should ideally be positioned at least two-thirds distal to the LCx and
2. The disc, which seals the anatomic ostium of the LAA.

**Fig. 19.**
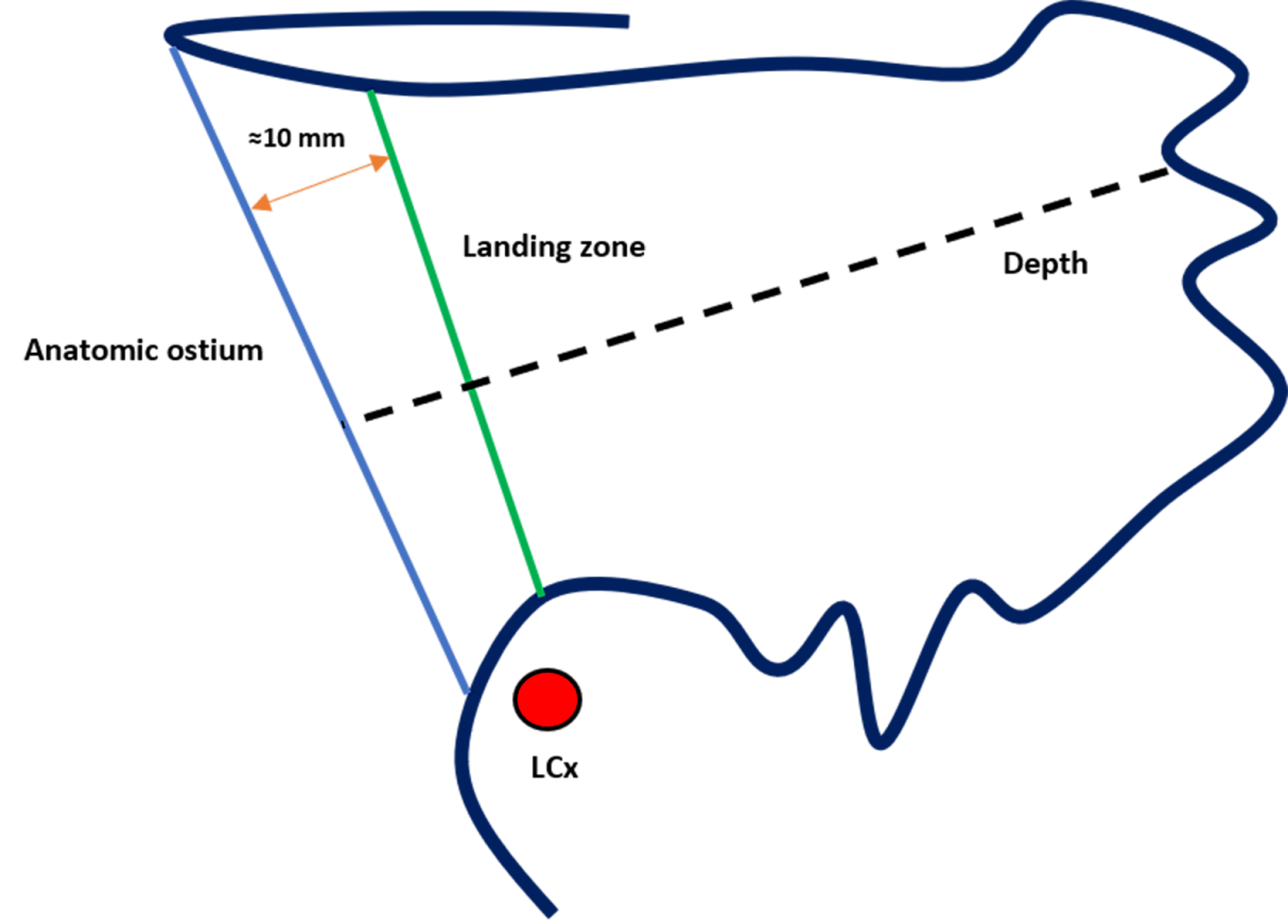
**Assessment of landing zone dimensions and depth for left atrial appendage closure (LAAC) with the Amulet device**. LCx, left circumflex artery.

Device sizing is based on the diameter of the landing zone or the functional ostium of the LAA. The functional ostium is measured approximately 10 mm distal to the anatomic ostium and perpendicular to the LAA wall. The depth is determined from a line drawn perpendicular to the anatomic ostium to the LAA roof along the expected axis of the device (Fig. 19) [65, 66].

Adequate depth is required to ensure full accommodation of the lobe: >10 mm for devices up to 22 mm and >12 mm for devices measuring 25–34 mm. Incorrect depth can lead to the following complications:

- Proximal lobe deployment increases the risk of embolization or incomplete ostial coverage due to inadequate disc retraction.
- Overly deep deployment may cause excessive lobe compression, disc prolapse into the LAA, or LAA perforation and thrombus formation.

#### 7.2.2 Access and Imaging

The TSP and access to the LAA follow the same technique described for the Watchman FLX™. The optimal fluoroscopic projection for Amulet implantation is RAO 30°/cranial 20°, corresponding to a TEE view of around 45°. This orientation allows clear visualization of the anatomic ostium, LSPV ridge, and LCx.

#### 7.2.3 Device Deployment

Amulet is deployed in three main steps, the first two for lobe release and the third for disc deployment.

**Fig. 20.**
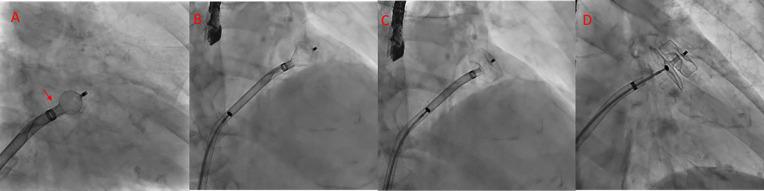
**Sequence of Amulet device deployment**. (A) Spherical configuration; in this position, the device can be safely rotated clockwise or counterclockwise. The platinum markers are located distal to the sheath’s radiopaque marker (arrowhead). (B) Triangular configuration; in this position, the device can be safely advanced. (C) The lobe is fully deployed. (D) Both the lobe and disc are fully deployed.

1. Initial lobe release: The sheath tip is positioned at the LCx level, and the lobe is partially unsheathed to form a ball-shaped configuration (Fig. 20). This allows safe clockwise or counterclockwise rotation to optimize the orientation of the device. If deeper placement is required, then further unsheathing converts the lobe into a triangular configuration, allowing safe advancement into the appendage. Once the optimal positioning is confirmed, the lobe is fully deployed by continued unsheathing while applying gentle forward pressure on the delivery cable.
2. Disc deployment: With gentle tension on the delivery cable, the disc is unsheathed to cover the LAA ostium. A tug test is performed by lightly retracting the system to confirm stability and proper alignment. The disc should adopt a concave configuration, indicating correct seating.
3. Repositioning (if required): If the disc shape or position is suboptimal, it can be recaptured by holding and retracting the delivery knob while gently pushing the sheath. Similarly, if lobe positioning is inadequate, the system can be recaptured into the ball configuration, confirmed under fluoroscopy when the platinum marker wires are distal to the sheath’s radiopaque marker (Fig. 20). This configuration protects the sheath tip from damage by the stabilizing wires.

#### 7.2.4 Final Assessment and Release

**Fig. 21.**
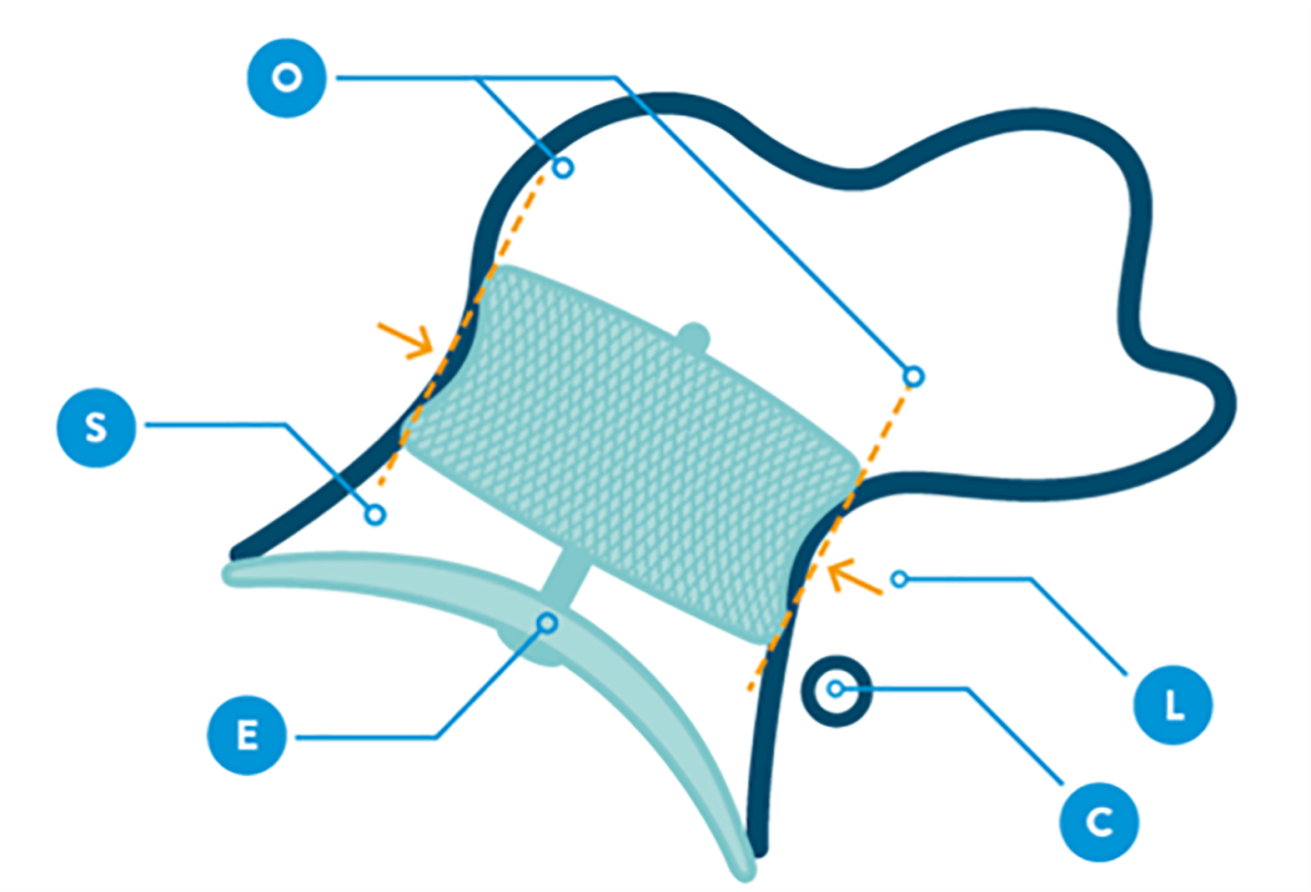
**CLOSE sign to verify device stability (image reproduced with permission from Abbott)**.

Before release, proper positioning should be verified using the CLOSE criteria (Fig. 21):

- C—Lobe positioned at least two-thirds distal to the LCx;
- L—Appropriate lobe compression;
- O—Orientation of the lobe aligned with the LAA neck axis;
- S—Visible separation between the lobe and disc;
- E—Elliptical or concave disc configuration;

Once all criteria are satisfied, the device is definitively released by first advancing the sheath toward the disc, followed by counterclockwise rotation of the delivery knob.

### 7.3 Special Considerations

Although LAAC should ideally be performed only after confirming the absence of LAA thrombus, certain exceptional clinical situations—such as persistent thrombus despite adequate anticoagulation or absolute contraindication to OAC—may warrant proceeding by using a partial deployment technique with disc-and-lobe devices, under continuous TEE guidance and without contrast injection. In this approach, the lobe is opened outside the LAA and carefully advanced into a proper position under TEE visualization, followed by disc deployment to achieve complete closure and minimize embolic risk. In this particular situation the use of a concomitant cerebral protection device is strongly recommended [67].

## 8. Postprocedural Antithrombotic Therapy: Evidence From Published Studies

The optimal postprocedural antithrombotic regimen following LAAC remains an area of active investigation and is influenced by the type of device implanted, patient comorbidities, and bleeding risk. The first randomized studies—PROTECT-AF and PREVAIL [20, 21] included patients without contraindications to long-term anticoagulation and compared LAAC with warfarin. In both trials, patients assigned to the device arm received warfarin plus aspirin for 45 days, followed by DAPT for 6 months and lifelong aspirin thereafter. However, this protocol is difficult to generalize to current practice, because most LAAC procedures today are performed in patients with absolute or relative contraindications to long-term anticoagulation, and DOACs have largely replaced warfarin as the standard of care.

The ASAP study [26], specifically enrolled patients ineligible for warfarin, mainly because of previous major bleeding. After Watchman™ implantation, all patients received DAPT for 6 months followed by lifelong aspirin. DRT occurred in 4% of patients, and the annual ischemic stroke rate was 1.7%, significantly lower than the expected rate based on CHADS_2_ scores. Although the sample size was relatively small (n = 150), this regimen appears reasonable for patients in whom long-term anticoagulation is contraindicated, recognizing that DRT remains a concern since even temporary resumption of OAC may pose substantial risk.

Similarly, the EWOLUTION registry [27] reported that 60% of patients received DAPT post-Watchman™ implantation, typically discontinued after 6 months. Other regimens included VKA (16%), DOAC (11%), single antiplatelet therapy (7%), and no antithrombotic therapy (6%). DRT occurred in 3.7% of patients and ischemic stroke in 1.1% of patients, with no correlation with the chosen regimen. Although heterogeneous, this real-world approach supports DAPT as a feasible option in patients unsuitable for OAC (73% of the cohort).

The PINNACLE FLX study evaluated the newer-generation Watchman FLX™ device in patients with AF who had an indication for OAC. Post-implantation, patients were treated with a DOAC (preferably apixaban or rivaroxaban) plus aspirin for at least 45 days. If TEE at 45 days confirmed adequate sealing (residual leak <5 mm), then DOAC therapy was discontinued and replaced with DAPT until 6 months post-implantation, followed by indefinite aspirin therapy. At 45 days, 96.2% of patients had discontinued the DOAC; major bleeding occurred in 3% of patients during that period. At the 1-year follow-up, seven patients had DRT. This strategy—evaluated in a population without OAC contraindication—aligns well with modern clinical practice and demonstrates the feasibility of a DOAC-based approach over traditional VKA regimens.

A sub-analysis of the SURPASS registry [68] including 53,878 patients compared outcomes according to different postprocedural strategies. At both 45 days and 6 months, DOAC monotherapy was associated with fewer major adverse events and major bleeding compared with DOAC + aspirin, supporting DOAC alone as a safe and effective regimen for patients eligible for anticoagulation after LAAC.

The ADALA randomized trial [69] directly compared low-dose DOAC therapy versus DAPT after LAAC. Despite its modest sample size, the study showed superior balance of efficacy—prevention of thromboembolism, SE, and DRT—and safety, with lower major bleeding rates in the DOAC group during the first three months. Importantly, 58.8% of enrolled patients had a prior major bleeding event, underscoring the clinical relevance of this finding.

### 8.1 Consensus Recommendations and Individualization of Therapy

The European Heart Rhythm Association/European Association of Percutaneous Cardiovascular Interventions (EHRA/EAPCI) expert consensus [25] emphasizes an individualized, risk-based approach to postprocedural antithrombotic therapy.

- Patients with a low bleeding risk undergoing Watchman™ implantation are given warfarin or a DOAC plus aspirin for up to 45 days. Once complete LAA occlusion is confirmed, OAC can be discontinued, and clopidogrel can be continued for up to 6 months, followed by aspirin indefinitely.
- Patients with a high bleeding risk undergoing Watchman™ implantation are given aspirin indefinitely plus clopidogrel for 1–6 months, depending on imaging confirmation of complete occlusion and absence of DRT.
- Patients undergoing Amulet implantation are given aspirin indefinitely combined with clopidogrel for 1–6 months, ensuring adequate occlusion and no evidence of DRT.

Ultimately, post-LAAC antithrombotic management should balance thromboembolic protection against bleeding risk, considering the device type, procedural findings, and patient comorbidities. Emerging evidence supports simplified DOAC-based regimens, although ongoing randomized trials will further define optimal strategies.

As a practical guide after LAAC, antithrombotic therapy should be individualized to balance the prevention of DRT with the risk of major bleeding. The choice of regimen depends on the patient’s bleeding and stroke risk, comorbidities, clinical condition, preferences, and the indication for LAAC. Discontinuation of OAC or antiplatelet therapy is appropriate only when no other clinical indications exist and imaging confirms the absence of significant peri-device leaks (>5 mm), device thrombus, or recent clinical events [70].

### 8.2 Postprocedural Antithrombotic Therapy: Insights From Ongoing Trials

The ANDES trial [71] is a randomized study enrolling patients eligible for short-term OAC. It compares 8 weeks of post-implant DOAC therapy with DAPT, with DRT assessed by TEE at 45 days. The results are expected to clarify the optimal early antithrombotic regimen, particularly regarding the prevention of DRT in patients who can safely receive short-term OAC.

The SAFE-LAAC trial [72] is evaluating the safety and efficacy of 30-day versus 6-month DAPT following LAAC. Additionally, it includes a non-randomized observational arm comparing complete discontinuation of antiplatelet therapy at 6 months versus continuation of long-term single antiplatelet therapy. This trial will help determine whether abbreviated DAPT or early antiplatelet discontinuation can safely reduce bleeding risk without increasing thromboembolic events.

The FADE-DRT trial [73] is a multicenter, randomized controlled study that incorporates genetic testing to identify clopidogrel responders and non-responders, thereby introducing a personalized approach to post-implant therapy. Three treatment arms are compared:

1. OAC for 6 weeks, followed by DAPT until 6 months, then aspirin monotherapy;
2. OAC for 6 weeks, followed by DAPT in clopidogrel responders or aspirin plus half-dose OAC in non-responders until 6 months, then aspirin monotherapy;
3. Half-dose DOAC for the entire follow-up period.

The primary endpoints are a composite of stroke, SE, and DRT at 1 year, as well as major bleeding at 1 year post-LAAC. This trial is particularly innovative for exploring genotype-guided antithrombotic therapy.

The ASPIRIN-LAAO trial [74] is a randomized controlled study conducted in patients undergoing LAAC with the Watchman™ device, comparing aspirin versus placebo beyond 6 months after implantation. During the initial 6 months, antithrombotic therapy is prescribed at the physician’s discretion. The primary endpoint is a composite of stroke, SE, CV or unexplained death, acute coronary syndrome, coronary or peripheral revascularization, and major bleeding. This trial aims to determine whether continuing aspirin beyond six months confers clinical benefit or, conversely, increases bleeding risk in patients without other indications for aspirin.

SIMPLAAFY [75] is an ongoing multicenter randomized trial aimed at evaluating the safety and efficacy of three different therapies following Watchman FLX™ Pro implantation: aspirin alone, a reduced dose of DOAC, or DAPT.

Collectively, these ongoing studies are expected to provide crucial evidence to guide individualized post-LAAC therapy, balancing the competing risks of thrombosis and bleeding and moving clinical practice toward more tailored antithrombotic strategies.

## 9. Conclusions and Future Perspectives

LAAC is a structural heart intervention that has become increasingly safe and effective for patients with AF with a moderate-to-high risk of thromboembolic events. Initially reserved for those with contraindications to long-term OAC, LAAC is now emerging as a valuable alternative in broader clinical scenarios—such as recurrent stroke despite adequate anticoagulation or in patients with CKD—where it may help mitigate both ischemic and bleeding risks. A meticulous preprocedural evaluation using TEE or CCT is essential to delineate the anatomic characteristics of the LAA and to identify challenging morphologies that may impact procedural strategy. Careful planning guided by advanced imaging plays a pivotal role in procedural success and patient safety.

Although complication rates have declined significantly over time due to greater operator experience and technical refinements, LAAC remains a preventive intervention, and any serious procedural complication can have major prognostic consequences. Therefore, adherence to optimal technique and comprehensive periprocedural management remains crucial.

There is currently no universally accepted postprocedural antithrombotic regimen, particularly given that LAAC is performed in both OAC-eligible and OAC-ineligible patients. The main challenge lies in managing patients with absolute or relative contraindications to anticoagulation, for whom the optimal therapeutic approach remains uncertain. A short course of a DOAC may be reasonable when anticoagulation is not absolutely contraindicated, whereas DAPT may serve as an alternative, at least until follow-up imaging confirms adequate device sealing and absence of DRT.

Ongoing and future trials assessing LAAC in various clinical contexts—such as its role as a substitute for OAC in eligible patients or its use in CKD—are expected to further broaden the indications for this therapy. In parallel, comparative studies of postprocedural antithrombotic strategies may help define the most effective and safest pharmacological approach across different patient subsets. Finally, advances in preprocedural planning, operator training, and device technology—including innovations aimed at minimizing thrombogenicity and procedural risk—are likely to enhance procedural outcomes and patient safety in the coming years, reinforcing LAAC as a cornerstone in stroke prevention for selected patients with AF.
